# Cell‐specific genome‐scale metabolic modeling of SARS‐CoV‐2‐infected lung to identify antiviral enzymes

**DOI:** 10.1002/2211-5463.13710

**Published:** 2023-09-30

**Authors:** Ke‐Lin Chen, Feng‐Sheng Wang

**Affiliations:** ^1^ Department of Chemical Engineering National Chung Cheng University Chiayi Taiwan

**Keywords:** antiviral target design, constraint‐based model, fuzzy optimization, genome‐scale metabolic model, hybrid differential evolution, multilevel optimization

## Abstract

Computational systems biology plays a key role in the discovery of suitable antiviral targets. We designed a cell‐specific, constraint‐based modeling technique for severe acute respiratory syndrome coronavirus 2 (SARS‐CoV‐2)‐infected lungs. We used the gene sequence of the alpha variant of SARS‐CoV‐2 to build a viral biomass reaction (VBR). We also used the mass proportion of lipids between the viral biomass and its host cell to estimate the stoichiometric coefficients of viral lipids in the reaction. We then integrated the VBR, the gene expression of the alpha variant of SARS‐CoV‐2, and the generic human metabolic network Recon3D to reconstruct a cell‐specific genome‐scale metabolic model. An antiviral target discovery (AVTD) platform was introduced using this model to identify therapeutic drug targets for combating COVID‐19. The AVTD platform not only identified antiviral genes for eliminating viral replication but also predicted side effects of treatments. Our computational results revealed that knocking out dihydroorotate dehydrogenase (DHODH) might reduce the synthesis rate of cytidine‐5′‐triphosphate and uridine‐5′‐triphosphate, which terminate the viral building blocks of DNA and RNA for SARS‐CoV‐2 replication. Our results also indicated that DHODH is a promising antiviral target that causes minor side effects, which is consistent with the results of recent reports. Moreover, we discovered that the genes that participate in the *de novo* biosynthesis of glycerophospholipids and ceramides become unidentifiable if the VBR does not involve the stoichiometry of lipids.

## Graphical abstract

The antiviral target discovery platform comprises two primary frameworks. First, we leverage RNA‐sequence expressions from host–virus cells and their host cells to reconstruct genome‐scale metabolic models tailored to each cell type. Following that, the second framework employs both models to identify potential antiviral targets using a fuzzy multiobjective hierarchical optimization process.

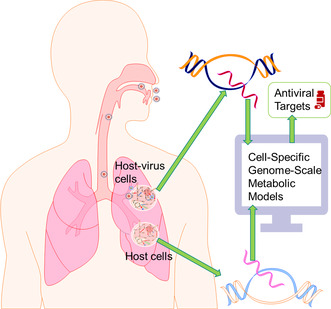

**Abbreviations**
AVTD: antiviral target discovery
BPGM: bisphosphoglycerate mutase
CAD: CAD protein
CEPT1: choline/ethanolaminephosphotransferase 1
CMPK1: UMP‐CMP kinase
CRLS1: cardiolipin synthase (CMP‐forming)
CTPS1: CTP synthase 1
DHODH: dihydroorotate dehydrogenase
ENO1: alpha‐enolase
FBA: flux balance analysis
FH: fumarate hydratase
GAPDH: glyceraldehyde‐3‐phosphate dehydrogenase
GMPR2: GMP reductase 2
GPR: gene protein reaction
GSMM: genome‐scale metabolic model
HMGCR: 3‐hydroxy‐3‐methylglutaryl‐coenzyme A reductase
HMGCS1: hydroxymethylglutaryl‐CoA synthase
HT: host
HV: host–virus
KDSR: 3‐ketodihydrosphingosine reductase
MDM: maximizing decision‐making
PGK1: phosphoglycerate kinase 1
PGS1: CDP‐diacylglycerol–glycerol‐3‐phosphate 3‐phosphatidyltransferase
PH: perturbed host
PLD2: phospholipase D2
PTDSS1: phosphatidylserine synthase 1
PTPMT1: phosphatidylglycerophosphatase and protein‐tyrosine phosphatase 1
RPIA: ribose‐5‐phosphate isomerase
SLC2A13: proton myoinositol cotransporter
SPTLC1: serine palmitoyltransferase 1
TR: treated host–virus
UFD: uniform flux distribution
UMPS: uridine 5′‐monophosphate synthase
VBGR: viral biomass growth rate
VBR: viral biomass reaction

Since its emergence in December 2019 [1], COVID‐19 has continued to pose a threat to humanity and has caused a substantial increase in global mortality as well as economic and social disruptions [2]. According to the WHO Coronavirus (COVID‐19) Dashboard (https://covid19.who.int/) [3], COVID‐19 has resulted in over 767 million infections and over 6.938 million deaths worldwide as of May 31, 2023. Vaccines authorized against COVID‐19 are currently widely available to prevent viral infection and limit the spread of the disease. According to the aforementioned dashboard, more than 13 billion vaccine doses have been administered as of May 31, 2023. However, only few antiviral drugs have been approved by the Food and Drug Administration (FDA) of the USA for the treatment of COVID‐19. Remdesivir was the first drug approved for treating COVID‐19 by the FDA. Ritonavir‐boosted nirmatrelvir (Paxlovid), molnupiravir, and certain monoclonal antibodies against severe acute respiratory syndrome coronavirus 2 (SARS‐CoV‐2) have been authorized for emergency use by the FDA for the treatment of COVID‐19. The Coronavirus Disease 2019 Treatment Guidelines, accessible at https://www.covid19treatmentguidelines.nih.gov/, provide comprehensive information on the treatment of COVID‐19. Many drug screening and repurposing techniques [4, 5, 6, 7, 8, 9, 10, 11, 12, 13] have also been developed to identify antiviral targets for the treatment of COVID‐19.

Flux balance analysis (FBA) is a constraint‐based modeling technique that has been widely used in the analysis of metabolic fluxes through a metabolic network. It has also been successfully used in fundamental research [14, 15, 16], oncogene inference [17, 18, 19, 20, 21, 22, 23], anticancer target discovery for the treatment of cancer metabolism [24, 25, 26, 27, 28, 29, 30], microbial engineering [31, 32, 33], and other research fields. Flux balance analysis (FBA) has been used to analyze the metabolic reprogramming behavior of viral infections [34, 35, 36]. Because viruses do not exhibit an independent metabolic system for replication, they depend entirely on their hosts. Experimental findings have revealed substantial metabolic flux changes in infected host cells compared with their host counterparts [34, 35, 36]. These metabolic alterations can be used to compare the differential expression of metabolites between a host–virus (HV) model and a host to identify potential therapeutic targets for the treatment of COVID‐19 [37, 38, 39, 40, 41, 42, 43, 44, 45, 46, 47]. To investigate the metabolic fluxes of an HV cell, a viral biomass reaction (VBR) must be constructed using the viral genome sequence. In previous studies, the VBR of the alpha variant of SARS‐CoV‐2 was incorporated into the iAB‐AMØ‐1410 human alveolar macrophage model [40, 42, 43, 44] and the Recon2.2 and Recon3D human genome‐scale metabolic models (GSMMs) [45, 46, 47, 48, 49] to examine the metabolic behavior of infected cells. However, based on a literature review, only a limited number of studies have endeavored to utilize a cell‐specific GSMM for identifying antiviral enzymes aimed at treating infected HV cells. Moreover, due to the scarcity of dynamic experimental data on viral envelopes, most studies have not addressed the stoichiometric information of viral lipids in the VBR.

The present study proposed the mass proportion of lipids between a viral biomass and its host cell to estimate the stoichiometric coefficients of viral lipids in a VBR. RNA‐seq expressions of SARS‐CoV‐2‐infected lungs were used to reconstruct cell‐specific GSMMs of HV cells and their host counterparts. Both models were then used to identify antiviral targets through an antiviral target discovery (AVTD) framework [47]. A VBR excluding viral lipids was also applied to the cell‐specific GSMMs to identify additional antiviral enzymes for comparison with the results obtained from the VBR lipid data.

## Materials and methods

The AVTD framework consisted of three parts: a VBR building block, a cell‐specific GSMM reconstruction module, and an antiviral target identification module. The gene and protein sequences of virus cells are used to construct the VBR, which is discussed in Constructing a VBR. The VBR is integrated with a human metabolic network Recon3D to form a universal network. The universal network and RNA‐seq expressions for host (HT) cells and virus‐infected cells are used to reconstruct cell‐specific GSMMs. The reconstruction module is discussed in Reconstruction of cell‐specific GSMM. We use fuzzy set theory and cell‐specific GSMMs to establish a fuzzy hierarchical multiobjective optimization framework for identifying antiviral targets. The framework is discussed in Discovery of antiviral targets.

### Constructing a VBR

Viruses do not replicate independently. Instead, they infect host cells to use these cells' metabolic protein synthesis pathways for reproduction. Viruses inject their genetic material into host cells, where it is replicated and packaged into new viruses. These new viruses then infect other hosts, continuing the cycle of viral replication. A key step in viral replication is the synthesis of a viral biomass within the HT cells, including structural proteins, lipids, and genetic material. The gene and protein sequences of the SARS‐CoV‐2 alpha (NC_045512), delta (MZ724506), and omicron (OL672836) variants can be downloaded from the National Center for Biotechnology Information (NCBI) website (https://www.ncbi.nlm.nih.gov/nuccore/), and the stoichiometric coefficients of their corresponding VBRs can be generated following the seven steps described by Aller *et al*. [50], Renz *et al*. [40], and Delattre *et al*. [45]. The approaches discussed in Refs [40, 45, 50] calculate the stoichiometric coefficient of H_2_O based on the number of molecules of H_2_O obtained from the hydrolysis of ATP. However, they do not account for the dehydration that occurs during nucleotide and protein condensation polymerization. Wang *et al*. [47] revised the stoichiometric calculation to address the water produced during the formation of this dehydration. However, most studies have not addressed the stoichiometric data of viral lipids in a VBR. In this study, we proposed the mass proportion of lipids between a viral biomass and its host cell to estimate the stoichiometric coefficients of viral lipids in a VBR. A VBR that includes lipids can be expressed as follows::

(1) $$\begin{array}{l} \sum_{N_{i}=\mathrm{CTP},\mathrm{GTP},\mathrm{UTP}}S_{N_{i}}N_{i}+\sum_{j=1}^{20}S_{A_{j}}A_{j}+S_{H_{2}O}H_{2}O+S_{\mathrm{ATP}}\mathrm{ATP}\\ \;+\;\alpha \sum_{p=1}^{9}S_{L_{p}}^{\mathrm{HT}}L_{p}\overset{\text{VBGR}}{\to }\text{Virus}‐\text{Biomass}\\ \;+\;S_{\mathrm{ADP}}\mathrm{ADP}+S_{P_{i}}P_{i}+S_{{\mathrm{PP}}_{i}}{\mathrm{PP}}_{i}\\ \;+S_{H^{+}}H^{+} \end{array}$$

where $S_{L_{p}}^{\mathrm{HT}}$ is the stoichiometric coefficient of lipids in the host cell, α is a mass ratio of lipids in the viral biomass relative to its host cell, and VBGR denotes as the viral biomass growth rate. The stoichiometric coefficients of nucleotides (*N*_*i*_), amino acids (*A*_*j*_), water, ATP, adenosine diphosphate (ADP), orthophosphate (*P*_*i*_), diphosphate (PP_*i*_), and proton (H^+^) are calculated as follows:

(2) $$\left\{\begin{array}{l} S_{N_{i}}=1000\frac{M_{N_{i}}^{\mathrm{Tot}}}{{\mathrm{MW}}_{\text{virus}}},\;\text{for}\;N_{i}=\mathrm{CTP},\mathrm{GTP}\;\text{and}\;\mathrm{UTP}\\ S_{A_{j}}=1000\frac{M_{A_{j}}^{\mathrm{Tot}}}{{\mathrm{MW}}_{\text{virus}}}\;\\ S_{H_{2}O}=1000\frac{\left(k_{\mathrm{ATP}}-1\right)\left(\sum M_{A_{j}}^{\mathrm{Tot}}-1\right)-M_{H_{2}O}^{\mathrm{Tot}}}{{\mathrm{MW}}_{\text{virus}}}\\ S_{\mathrm{ADP}}=S_{P_{i}}=S_{H^{+}}=1000\frac{k_{\mathrm{ATP}}\left(\sum M_{A_{j}}^{\mathrm{Tot}}-1\right)}{{\mathrm{MW}}_{\text{virus}}}\\ S_{\mathrm{ATP}}=1000\left(\frac{\sum M_{\mathrm{ATP}}^{\mathrm{Tot}}+k_{\mathrm{ATP}}\left(\sum M_{A_{j}}^{\mathrm{Tot}}-1\right)}{{\mathrm{MW}}_{\text{virus}}}\right)\\ S_{{\mathrm{PP}}_{i}}=1000\frac{M_{{\mathrm{PP}}_{i}}^{\mathrm{Tot}}}{{\mathrm{MW}}_{\text{virus}}}\\ S_{A_{j}}=1000\frac{M_{A_{j}}^{\mathrm{Tot}}}{{\mathrm{MW}}_{\text{virus}}} \end{array}\right.$$

Here, the total moles, namely the $M_{N_{i}}^{\mathrm{Tot}}$, $M_{A_{j}}^{\mathrm{Tot}}$, and $M_{{\mathrm{PP}}_{i}}^{\mathrm{Tot}}$ values of the *i*th nucleotide, *j*th amino acid and PPi molecule, respectively, are calculated using the molecule counts of the gene and protein sequences and expressed as follows:

(3) $$\left\{\begin{array}{l} M_{N_{i}}^{\mathrm{Tot}}=C_{G}\left(F_{N_{i}}^{G}+F_{N_{i}}^{R}\right),N_{i}=\mathrm{ATP},\mathrm{CTP},\mathrm{GTP}\;\text{and}\;\mathrm{UTP}\\ M_{{\mathrm{PP}}_{i}}^{\mathrm{Tot}}=M_{H_{2}O}^{\mathrm{Tot}}=k_{{\mathrm{PP}}_{i}}C_{G}\left(\left(\sum_{i}F_{N_{i}}^{G}-1\right)+\left(\sum_{i}F_{N_{i}}^{R}-1\right)\right)\\ M_{A_{j}}^{\mathrm{Tot}}=\sum_{k}C_{{\mathrm{SP}}_{k}}F_{A_{j}}^{{\mathrm{SP}}_{k}}+\sum_{k}C_{{\mathrm{NP}}_{k}}F_{A_{j}}^{{\mathrm{NP}}_{k}} \end{array}\right.$$

where the frequency $F_{N_{i}}^{G}$ in the viral genome and the frequency $F_{N_{i}}^{R}$ in the replication intermediate of each nucleotide *N*_*i*_ are calculated using the viral gene sequence retrieved from the NCBI database (https://www.ncbi.nlm.nih.gov/nuccore/); *C*_G_, $C_{{\mathrm{SP}}_{k}}$ and $C_{{\mathrm{NP}}_{k}}$ are the copy numbers of the gene sequence, the *k*th structural protein and the *k*th nonstructural protein, respectively. The frequency $F_{A_{j}}^{{\mathrm{SP}}_{k}}$ of the amino acid *A*_*j*_ in the *k*th structural protein and the frequency $F_{A_{j}}^{{\mathrm{NP}}_{k}}$ of the amino acid *A*_*j*_ in the *k*th nonstructural protein are calculated using the viral protein sequence from the NCBI database.

Dynamic experimental data on viral envelopes are scarce. Renz *et al*. [41] used five lipids, namely phosphatidylcholine, phosphatidylethanolamine, phosphatidylinositol, phosphatidylserine, and cholesterol, with various stoichiometric coefficients from a macrophage biomass function factorized with a multiplication parameter to determine the effects of these lipids on the VBR. However, they discovered that with this factorization, the stoichiometric coefficients of lipids did not exhibit a unique representation in the VBR. In this study, we used the mass proportion of lipids between the viral biomass and its host cell to estimate the stoichiometric coefficients of viral lipids in the VBR, as expressed in Eqn (1). The biomass reaction of the host (HT) cells is expressed as follows:

(4) $$\begin{array}{l} \sum_{N_{i}=\mathrm{CTP},\mathrm{GTP},\mathrm{UTP}}S_{N_{i}}^{\mathrm{HT}}N_{i}+\sum_{j=1}^{20}S_{A_{j}}^{\mathrm{HT}}A_{j}+S_{H_{2}O}^{\mathrm{HT}}H_{2}O+S_{\mathrm{ATP}}^{\mathrm{HT}}\mathrm{ATP}+\sum_{p}^{P}S_{L_{p}}^{\mathrm{HT}}L_{p}\overset{\text{VHOST}}{\to }\text{Host}\_\text{Biomass}+S_{\mathrm{ADP}}^{\mathrm{HT}}\mathrm{ADP}+S_{P_{i}}^{\mathrm{HT}}P_{i}+S_{{\mathrm{PP}}_{i}}^{\mathrm{HT}}{\mathrm{PP}}_{i}+S_{H^{+}}^{\mathrm{HT}}H^{+} \end{array}$$

where the stoichiometric coefficients, namely $S_{N_{i}}^{\mathrm{HT}}$, $S_{A_{j}}^{\mathrm{HT}}$, …, $S_{H^{+}}^{\mathrm{HT}}$, of the host biomass reaction are obtained from a human GSMM. We used the biomass reaction in Eqn (4) to calculate the ratio of the total mass of lipids relative to the mass of the host cells as follows:

(5) $$\text{Ratio}=\frac{\sum_{p=1}^{8}S_{L_{p}}^{\mathrm{HT}}L_{p}}{\text{Host}\_\text{biomass}}=\frac{\sum_{p=1}^{8}S_{L_{p}}^{\mathrm{HT}}L_{p}}{\left(\begin{array}{l} \sum_{N_{i}=\mathrm{CTP},\mathrm{GTP},\mathrm{UTP}}S_{N_{i}}^{\mathrm{HT}}N_{i}+\sum_{j=1}^{20}S_{A_{j}}^{\mathrm{HT}}A_{j}+S_{H_{2}O}^{\mathrm{HT}}H_{2}O+S_{\mathrm{ATP}}^{\mathrm{HT}}\mathrm{ATP}+\sum_{p=1}^{8}S_{L_{p}}^{\mathrm{HT}}L_{p}-\\ \left(S_{\mathrm{ADP}}^{\mathrm{HT}}\mathrm{ADP}+S_{P_{i}}^{\mathrm{HT}}P_{i}+S_{{\mathrm{PP}}_{i}}^{\mathrm{HT}}{\mathrm{PP}}_{i}+S_{H^{+}}^{\mathrm{HT}}H^{+}\right) \end{array}\right)}$$

where the metabolite species, *N*_*i*_, *A*_*j*_, …, *H* ^+^, in Eqn (5) denote the corresponding molecular weights. Subsequently, we calculated the mass ratio of lipids relative to the biomass of the HV cell and assumed that the ratios for the HT cell and HV cell were identical. The mass ratio of lipids in the viral biomass relative to its host cell (α) is calculated as follows::

(6) $$\alpha =\frac{\text{Ratio}}{\left(1-\text{Ratio}\right)}\frac{\left(\begin{array}{l} \sum_{N_{i}=\mathrm{CTP},\mathrm{GTP},\mathrm{UTP}}S_{N_{i}}N_{i}+\sum_{j=1}^{20}S_{A_{j}}A_{j}+S_{H_{2}O}H_{2}O+S_{\mathrm{ATP}}\mathrm{ATP}-\\ \left(S_{\mathrm{ADP}}\mathrm{ADP}+S_{P_{i}}P_{i}+S_{{\mathrm{PP}}_{i}}{\mathrm{PP}}_{i}+S_{H^{+}}H^{+}\right) \end{array}\right)}{\sum_{p=1}^{8}S_{L_{p}}^{\mathrm{HT}}L_{p}}$$

### Reconstruction of cell‐specific GSMM

**Fig. 1:**
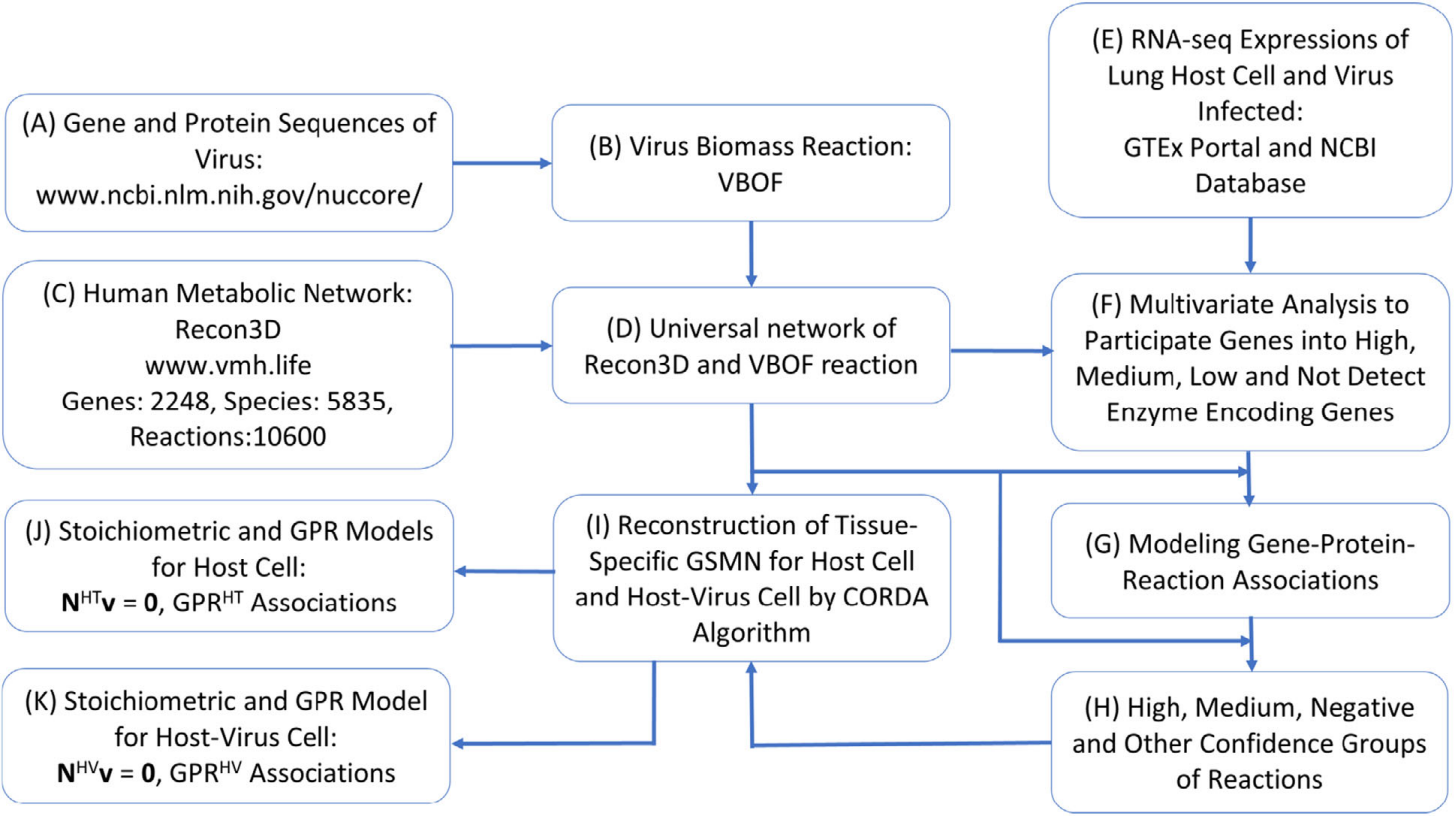
Reconstruction of cell‐specific GSMMs for the lung host (HT) cell and HV cell.

Figure 1 depicts the reconstruction of cell‐specific GSMMs of SARS‐CoV‐2‐infected lungs. In Step A, the gene and protein sequences of the alpha variant of SARS‐CoV‐2 were downloaded from the NCBI database (https://www.ncbi.nlm.nih.gov/nuccore/). In Step B, each gene and protein sequences were used to construct the corresponding VBR, as discussed in the previous subsection. In Step C, a human metabolic network Recon3D was downloaded from the Virtual Metabolic Human database (http://www.vmh.life/). Recon3D is a generic genome‐scale human metabolic network that consists of 2248 enzyme‐encoding genes, 5835 metabolite species, and 10 600 reactions. In Step D, Recon3D was integrated into the VBR to form a universal network for reconstructing a cell‐specific GSMM. In Step E, 578 samples of RNA‐seq expression for healthy lung cells [denoted as host (HT) cells] were downloaded from the Genotype‐Tissue Expression database (https://gtexportal.org/home/datasets), and the RNA‐seq expressions of 52 samples of SARS‐CoV‐2‐infected lungs (denoted as HV cells) were accessed from the NCBI database (https://www.ncbi.nlm.nih.gov/geo/query/acc.cgi?acc=GSE150316). In Step F, we utilized several statistical methods, including quantile normalization, averaging, confidence intervals, and quartiles, to analyze the RNA‐seq expressions of both healthy and infected samples. This analysis aimed to classify the enzyme‐encoding genes into four distinct groups: high, medium, low, and not detect. For additional details on the procedures of Step F, please refer to our previous studies [19, 21, 28, 29]. In Step G, the universal network and four groups of enzyme‐encoding genes were used to construct gene protein reaction (GPR) associations. In Step H, these GPR associations were used to classify the reactions in the universal network into four confidence reaction groups. In Step I, the four reaction groups and universal network were processed using the cost‐optimization reaction dependency assessment (CORDA) algorithm [51] to reconstruct cell‐specific GSMMs for the HT cell (Step J) and HV cell (Step K).

In this study, a reduced GPR association was established on the AVTD platform to avoid the superfluous computations in evolutionary optimization procedures. Gene protein reaction (GPR) associations are typically implemented as Boolean rules, which link metabolic reactions in stoichiometric models to gene‐encoded enzymes in cells. The pruning procedures discussed in our previous study [29] were used to delete the duplicate enzymes in the network to avoid the use of excessive computational steps when solving the AVTD problem.

### Discovery of antiviral targets

The AVTD framework is a fuzzy multiobjective hierarchical optimization problem that mimics a wet‐laboratory experiment to identify targets for treatment [47]. Table 1 presents the design concept of the AVTD framework. The outer optimization problem has four goals to evaluate the performance of each identified antiviral target. The primary goal is to minimize VBGR in treated HV cells (TR cells), signifying a candidate target's ability to block viral replication and growth. An antiviral target may interfere with host cells (perturbed HT cells, denoted as PH cells). The second goal aims to restore cell viability of TR cells and maintain PH cells as close as possible to a healthy state. This objective is evaluated by maximizing the ATP production rate for TR cells and PH cells. This objective is evaluated by maximizing the ATP production rate for TR cells and PH cells. To compare the metabolic distributions of TR cells and PH cells with the standard levels of HV and HT cells, which are referred to as metabolic templates and typically obtained from clinical data, over 5000 fluxes and metabolite flow rates are required. However, to date, a genome‐scale standard is not available. Therefore, in this study, we computed the metabolic distributions of HV and HT cells to serve as their respective metabolic templates.

**Table 1:** Hierarchical optimization framework of the adopted AVTD for identifying antiviral targets on the basis of four objectives.

Objectives in the outer optimization problem: To minimize the viral biomass growth rate (VBGR) of HV cells and the biomass growth rate of the host and HV cells with the target treatment To maximize the ATP production rate for treated HV cells and perturbed host cells during treatment To evaluate the metabolic flux patterns for treated HV cells and perturbed HT cells as similar to the HT template as possible To evaluate the metabolic flux patterns for treated HV cells and perturbed HT cells as dissimilar to the HV template as possible
Constraint‐based models in the inner optimization problems: FBA and UFD problems for the treated HV cells FBA and UFD problems for the perturbed HT cells

The third goal is to use fuzzy similarity to evaluate the extent of metabolic perturbation similarity between the metabolic patterns of TR cells and PH cells with respect to the metabolic template of HT cells. These metabolic patterns are defined as the distributions of fluxes and metabolite flow rates for TR cells and PH cells, respectively. The fourth goal is to use fuzzy dissimilarity to assess the extent of metabolic perturbation difference between the metabolic patterns of TR cells and PH cells with respect to the metabolic template of HV cells. We defined a metabolic deviation grade using two‐sided membership functions to evaluate fuzzy similarity and dissimilarity. A metabolic deviation grade, analogous to a satisfactory grade, can be used to evaluate the side effects of treatment. A higher metabolic deviation grade indicates a lower occurrence of side effects resulting from the treatment.

**Fig. 2:**
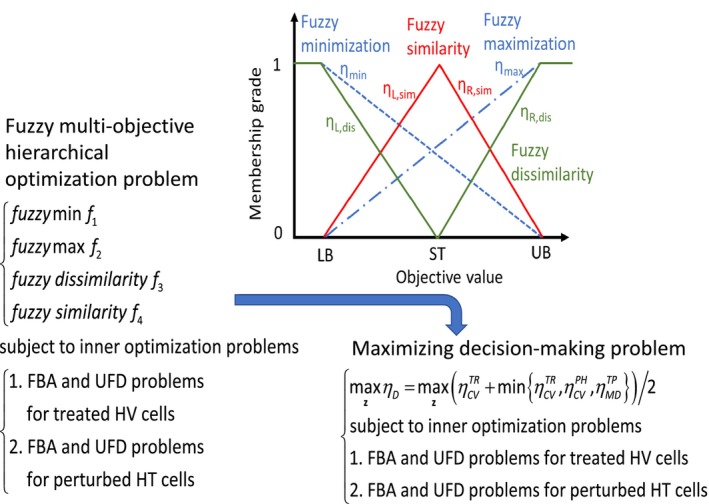
Transformation of a fuzzy multiobjective hierarchical optimization problem into an MDM problem through fuzzy membership functions. The lower bound (LB), upper bound (UB), and standard value (ST) are provided by the user. These values can be obtained from clinical data (if available); otherwise, they can be estimated using HT and HV templates. One‐sided linear membership functions (dashed and dash‐dotted lines) are used to evaluate fuzzy minimization and maximization. Two‐sided linear membership functions (red and green lines) are used to evaluate fuzzy similarity and dissimilarity.

Fuzzy set theory is used to transform AVTD problems into a maximizing decision‐making (MDM) problem to derive Pareto solutions (Fig. 2). The MDM problem can be expressed as follows:

(7) $$\left\{\begin{array}{l} \max_{z}\eta_{D}=\max_{z}\left(\eta_{\mathrm{CV}}^{\mathrm{TR}}+\min\left\{\eta_{\mathrm{CV}}^{\mathrm{TR}},\eta_{\mathrm{CV}}^{\mathrm{PH}},\eta_{\mathrm{MD}}^{\mathrm{TP}}\right\}\right)/2\\ \text{subject to inner optimization problems}\\ 1.\mathrm{FBA}\;\text{and}\;\mathrm{UFD}\;\text{problems for treated}\;\mathrm{HV}\;\text{cells}\\ 2.\mathrm{FBA}\;\text{and}\;\mathrm{UFD}\;\text{problems for perturbed}\;\mathrm{HT}\;\text{cells} \end{array}\right.$$

where $\eta_{\mathrm{CV}}^{\mathrm{TR}}$, $\eta_{\mathrm{CV}}^{\mathrm{PH}}$, and $\eta_{\mathrm{MD}}^{\mathrm{TP}}$ are as the cell viability grade of the treated HV (denoted as TR) model, the cell viability grade of the perturbed HT (denoted as PH) model, and the metabolic deviation grade of the TR and PH models relative to their corresponding templates, respectively. For additional details on these grades and the optimality of transformation between fuzzy multiobjective hierarchical optimization problems and MDM problems, please refer to our previous study [47]. An MDM problem is a challenging optimization problem that is difficult to solve directly using commercially available software [52, 53]. It is high‐dimensional, bilevel, and mixed‐integer linear, which makes it NP‐hard. However, there are a number of approximation algorithms and heuristics that can be used to find near‐optimal solutions. In this study, we used the nested hybrid differential evolution (NHDE) algorithm to solve the MDM problem. NHDE is a parallel direct search procedure that is an extended version of hybrid differential evolution [54]. A detailed description of the AVTD framework and its computational procedures is provided in Table S1.

## Results and Discussion

### Reconstructed cell‐specific models

**Fig. 3:**
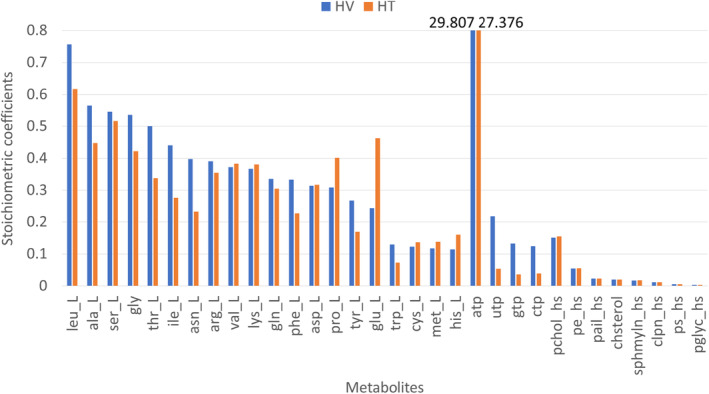
Stoichiometric coefficients for each metabolite of the viral biomass reaction in the HV and the biomass reaction in the HT models.

The generic human GSMM Recon3D was downloaded from the Virtual Metabolic Human database (http://www.vmh.life) to combine with the designed VBR to form a universal human GSMM. The universal network incorporated with the RNA‐seq expression data for the HT and HV cells was used to reconstruct cell‐specific GSMMs for the HT and HV cells, respectively. The stoichiometric coefficient of each metabolite of the VBR in the HV model was compared with that of the biomass reaction in the HT model (Fig. 3). The results showed that the ATP of the VBR had the highest stoichiometric coefficient in the HV model, with a value of 29.807. The biomass reaction had the highest stoichiometric coefficient in the HT model, with a value of 27.376. In addition, the stoichiometric coefficients of uridine‐5′‐triphosphate (UTP), guanosine‐5′‐triphosphate (GTP), and cytidine‐5′‐triphosphate (CTP) in the HV model were greater than those in the HT model. However, the stoichiometric coefficients of amino acids, l‐glutamate (glu_L), l‐proline (pro_L), and l‐histidine (his_L) in the HV model were smaller than those in the HT model. By contrast, the stoichiometric coefficients of l‐leucine (leu_L), l‐alanine (ala_L), glycine (gly), l‐asparagine (asn_L), l‐phenylalanine (phe_L), l‐tyrosine (tyr_L), and l‐tryptophan (trp_L) were greater than those of in the HT model.

**Fig. 4:**
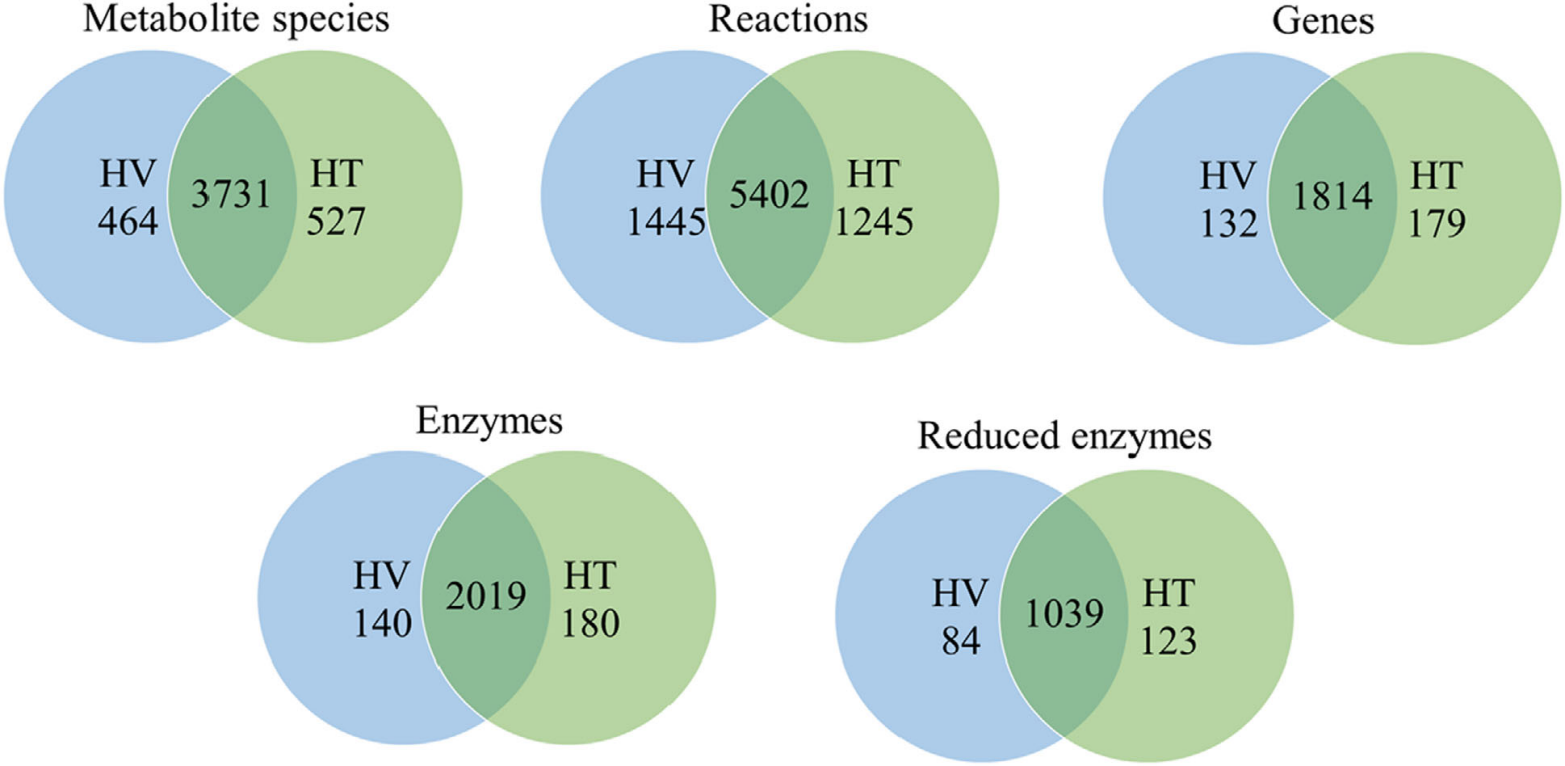
Comparison of the metabolic network data of the HV and HT models reconstructed using the CORDA algorithm. The values shown in the overlapping regions denote the numbers of identical metabolite species, reactions, genes, enzymes, and feasible enzymes in these models.

The universal network consisted of 5835 metabolites, 10 601 reactions, 2248 genes, and 2426 gene‐encoded enzymes. Some of the enzymes in this network were used to regulate the same reactions. To construct 1375 reduced enzymes and avoid superfluous computation steps while solving the MDM problem, we used the pruning procedures discussed in [29] to delete duplicate enzymes from the network. Figure 4 shows the numbers of metabolite species, reactions, genes, and encoded enzymes in the reconstructed cell‐specific GSMMs for HT and HV cells. As indicated by the overlapping regions in Fig. 4, both models shared numerous similarities in terms of metabolite species, reactions, genes, and enzymes. The cell‐specific GSMMs for HT cells can be found in Tables S3 and S4, while those for HV cells are available in Tables S5 and S6.

### One antiviral target

To identify optimal antiviral enzymes, the NHDE algorithm [28, 29, 55] was used to solve the MDM problem expressed in Eqn (7). This algorithm was run several times to identify a set of single antiviral targets with the highest fitness among 1123 reduced enzymes. A total of 23 antiviral targets were identified (Table 2). These targets could reduce the viral biomass growth rate (i.e., VBGR) from 15% (VBGR = 1.2736) to 100%. According to the reduced GPR association, six genes were duplicated, two genes had the same isozymes, and three genes formed a complex enzyme (Table 2). In wet‐laboratory experiments, reduced GPR associations allow for the simultaneous downregulation and deletion of all replicated genes.

**Table 2:** One‐target enzymes responsible for the reduction of the viral biomass growth rate within the AVTD framework. The symbols ↓ and Δ refer to the downregulation and knockout, respectively, of an enzyme. The superscripts ♣ and ♥ indicate that an enzyme has duplicate enzymes and isozymes, respectively, in the model. * indicates a complex enzyme. VBR, VBGR, and VATP represent the viral biomass reaction (VBR) and its corresponding biomass growth rate, and ATP production rate of treated HV cells.

Gene	Regulation	VBR includes the stoichiometry of lipids				VBR excludes the stoichiometry of lipids				Participated pathway
		VBGR	VATP	$\eta_{\mathrm{CV}}^{\mathrm{TR}}$	$\eta_{\mathrm{MD}}^{\mathrm{TP}}$	VBGR	VATP	$\eta_{\mathrm{CV}}^{\mathrm{TR}}$	$\eta_{\mathrm{MD}}^{\mathrm{TP}}$	
DHODH	Δ or ↓	0	3.6986	0.5487	0.5696	0	3.6986	0.5487	0.5181	Pyrimidine metabolism
(CTPS1, CTPS2)^♣^	Δ or ↓	0	3.6986	0.5487	0.5378	0	3.6986	0.5487	0.531	UTP and CTP Dephosphorylation I
KDSR	Δ or ↓	0	7.3604	0.5968	0.5373	1.5	21.3908	0.2816	0.3083	Sphingolipid biosynthesis
(SPTLC1, SPTLC2, SPTLC3)*	Δ or ↓	0	7.3604	0.5968	0.5373	1.5	21.3908	0.2816	0.3083	Sphingolipid biosynthesis
PTDSS1	Δ	0	3.6986	0.5487	0.5348	1.5	21.3908	0.2815	0.3224	Glycerophospholipid biosynthesis
UMPS	Δ	0	3.6986	0.5487	0.5301	0	3.6986	0.5487	0.4956	Pyrimidine metabolism
CAD	Δ	0	3.6986	0.5487	0.5254	0	3.6986	0.5487	0.5068	Pyrimidine metabolism
SLC2A13	Δ	0	3.6986	0.5487	0.5242	1.5	21.3908	0.2815	0.3268	Nuclear Receptors Meta‐pathway
CRLS1	Δ or ↓	0	3.6986	0.5487	0.5165	1.5	21.3908	0.2816	0.3307	Cardiolipin biosynthesis II
PGS1	Δ or ↓	0	36.802	0.9842	0.5124	1.5	21.3908	0.2816	0.3148	Cardiolipin biosynthesis II
PTPMT1	Δ or ↓	0	36.802	0.9842	0.5042	1.5	21.3908	0.2815	0.3083	Cardiolipin biosynthesis II
CMPK1	Δ	0	3.6986	0.5487	0.4206	0	3.6986	0.5487	0.4093	Remdesivir Pathway
(PLD2, PLD1, PLD3, PLD6)^♥^	↓	0.0013	38	0.9996	0.3255	0.0096	38	0.9968	0.4113	Alpha‐synuclein signaling
FH	↓	0.0033	38	0.9989	0.3236	0.0063	38	0.9979	0.4083	TCA cycle
HMGCS1	↓	0.0324	38	0.9892	0.3227	0.0236	38	0.9921	0.3468	Cholesterol biosynthesis
(GMPR2, GMPR)^♣^	↓	0.0009	38	0.9997	0.3207	0.0042	38	0.9986	0.3911	Nucleotide salvage
HMGCR	↓	0.0033	38	0.9989	0.2971	0.0078	38	0.9974	0.3122	Cholesterol biosynthesis
RPIA	Δ	0.3952	11.7889	0.5234	0.4084	0.3952	11.3205	0.5172	0.4174	Pentose phosphate pathway
(ENO1, ENO2, ENO3)^♣^	Δ	0.6676	38	0.7775	0.3239	0.6814	38	0.7729	0.3345	Glycolysis
(PGK1, PGK2, MIA3)^♣^	Δ	0.6676	38	0.7775	0.3173	0.6814	38	0.7729	0.4115	Glycolysis
(BPGM, PGAM1, PGAM2)^♣^	Δ	0.6676	38	0.7775	0.3115	0.6814	38	0.7729	0.3552	Glycolysis
(GAPDH, GPADHS)^♣^	Δ or ↓	0.6676	38	0.7775	0.2935	0.0006	38	0.9998	0.3394	Glycolysis
(CEPT1, SELENOI)^♥^	Δ	1.2736	21.5939	0.3596	0.3297	1.5	21.3908	0.2815	0.324	Phosphatidylcholine biosynthesis I

**Fig. 5:**
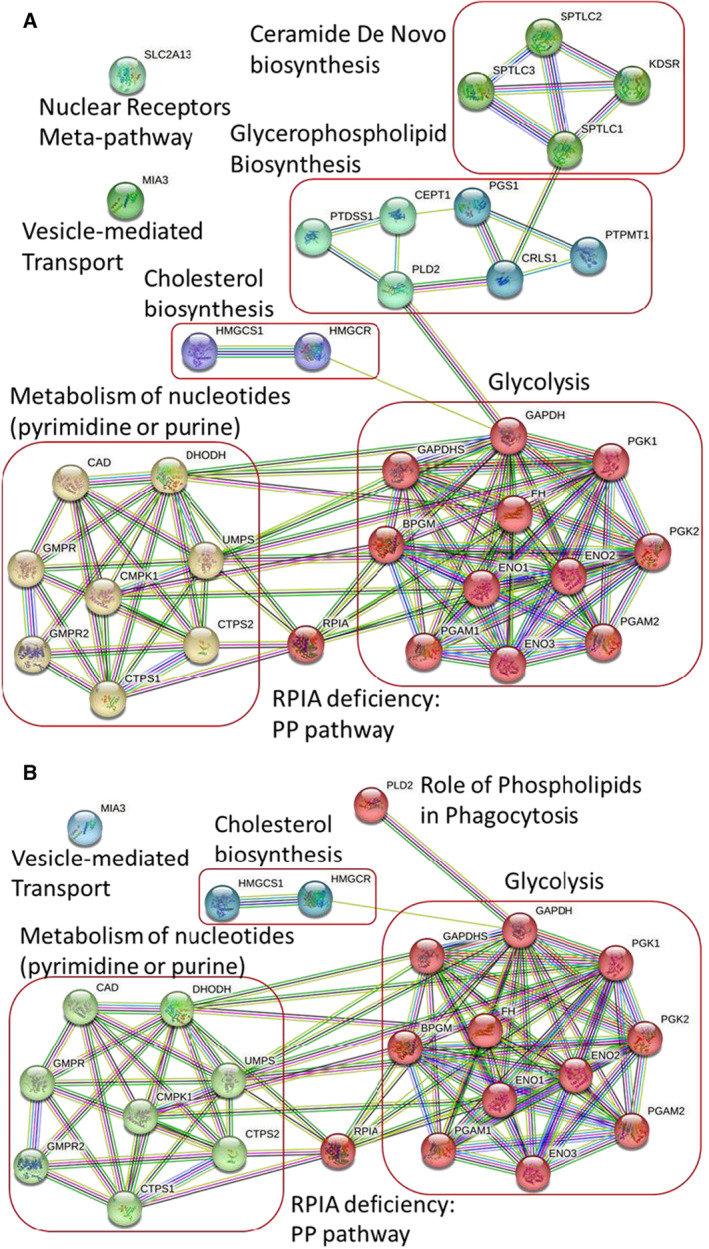
PPIs of identified genes: (A) VBOF with viral lipids and (B) VBOF without viral lipids.

We used STRING (https://string‐db.org/) and GeneCards (https://www.genecards.org/) to investigate the protein–protein interaction (PPI) networks encoded by the 23 aforementioned genes (Fig. 5A). Eight genes, including two duplicate genes, are involved in the metabolism of the nucleotides responsible for pyrimidine and purine metabolism. The downregulation or knockout of one of these genes might reduce the synthesis of nucleotides that serve as the basic building blocks of RNA and DNA, thereby reducing the VBGR (Table 2). Six genes are involved in the biosynthesis of glycerophospholipids, which serve as precursors of lipids and are regarded as essential structural and functional components of biological membranes. Four genes are involved in the *de novo* biosynthesis of ceramides, which serve as a metabolic hub in sphingolipid biosynthesis and catabolism. Ceramide *de novo* biosynthesis is a salvage pathway of glycosphingolipids (Fig. 5A). Therefore, the blockage of a gene in the glycerophospholipid or ceramide *de novo* biosynthesis pathway might reduce the synthesis of lipids necessary for the viral membrane, thereby reducing the VBGR (Table 2). Eleven genes are involved in glycolysis and glycosaminoglycan metabolism (Fig. 5A). Glycolysis is a metabolic pathway that converts glucose into pyruvate. Glycosaminoglycans play a crucial role in the regulation of cell growth and proliferation in the extracellular matrix (Table 2). Two genes are involved in the biosynthesis of cholesterol, which is used as a building block in the cell membrane (Fig. 5A).

**Fig. 6:**
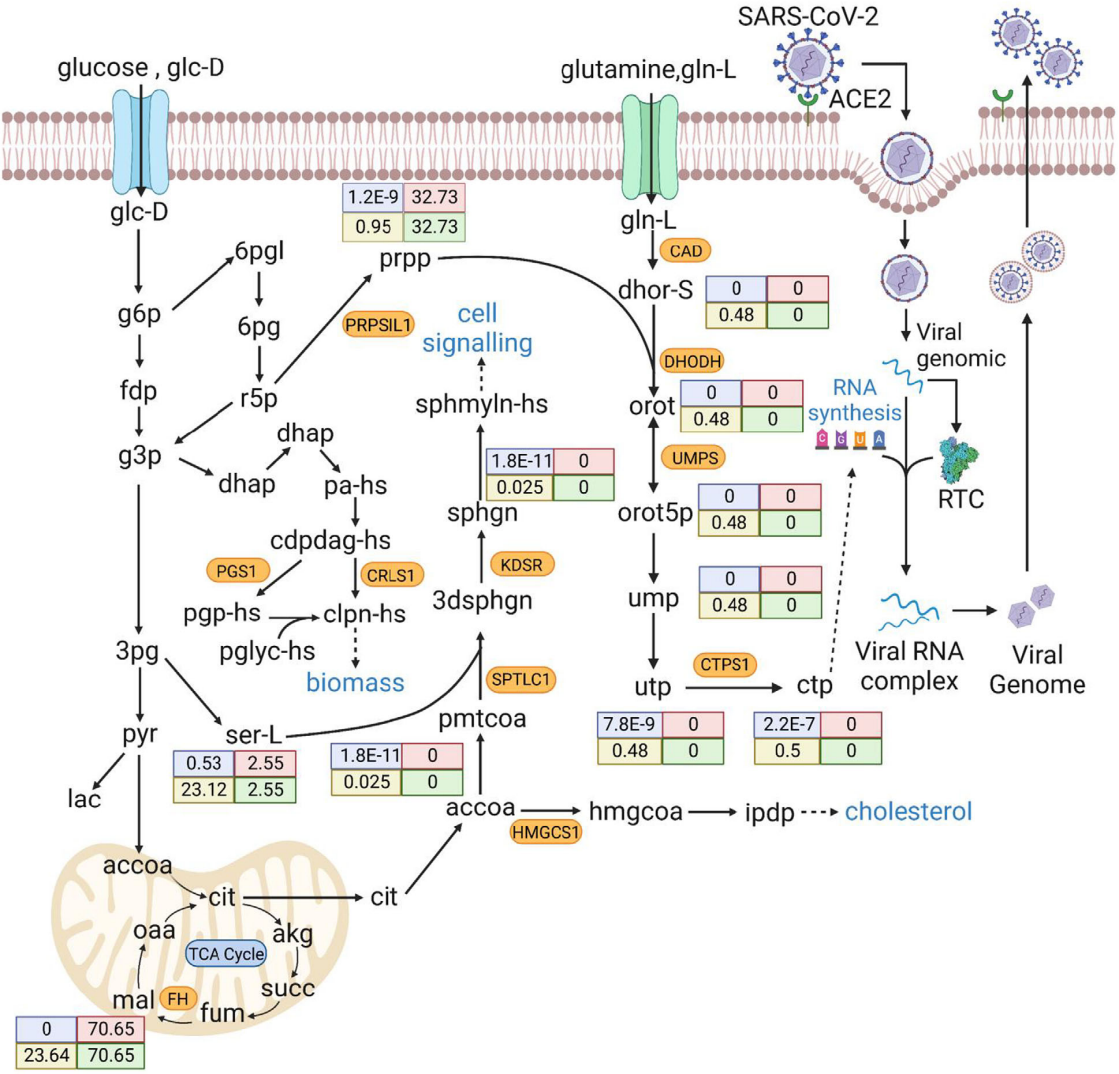
Metabolite flow rates of HV and HT cells and distributions modulated by DHODH. The first row in each box shows the metabolite flow rates of treated HV and perturbed HT cells modulated by DHODH, and the second row shows the metabolite flow rates of HV and HT templates. This figure was generated by BioRender.com.

Dihydroorotate dehydrogenase (DHODH) is a one‐target gene involved in the metabolism of nucleotides (Table 2). It catalyzes the oxidation of dihydroorotate to orotate (Orot) by using ubiquinone as an electron acceptor. DHODH is also involved in the metabolism of pyrimidine and purine, which are essential building blocks for DNA and RNA. Figure 6 depicts the metabolic pathway modulated by DHODH. In this study, the knockout of DHODH in HV cells reduced the synthesis rates of Orot, CTP, and UTP, which serve as viral building blocks of DNA and RNA for SARS‐CoV‐2 replication, from 0.48 to 0 mmol·gDW^−1^·h^−1^, from 0.5 to 2.72 × 10^−7^ mmol·gDW^−1^·h^−1^, and from 0.48 to 3.74 × 10^−7^ mmol·gDW^−1^·h^−1^, respectively. Therefore, the knockout of DHODH gene diminished the cell growth rate of treated HV cells. However, the maximum ATP production rate was 3.6986 mmol·gDW^−1^·h^−1^. Therefore, the cell viability grade of treated HV cells reached 0.5487 mmol·gDW^−1^·h^−1^ (Table 2). Within the AVTD framework, we hypothesized that the treatment process might induce metabolic perturbations in HT cells. Metabolic perturbations can serve as indicators to assess the level of side effects caused by the treatment. Hence, we calculated the metabolic deviation grade to evaluate the metabolic perturbations in TR cells and PH cells concerning the HT and HV templates, resulting in a score of 0.5696. This score indicates a satisfactory grade of 56.96% in predicting the occurrence of side effects.

DHODH has been implicated in a variety of diseases, including cancer and immunological disorders such as acute myeloid leukemia, rheumatoid arthritis, and multiple sclerosis [56, 57, 58]. Recently, DHODH has also emerged as a potential target for the treatment of COVID‐19, as it may interfere with the viral replication and host response [56, 57, 58, 59, 60]. Several drugs that inhibit DHODH, such as PTC299 [56, 57], teriflunomide [57], and leflunomide [58], are already approved for other indications and could be repurposed for COVID‐19 therapy [56, 57, 58, 59, 60]. Our computational results also revealed that the DHODH inhibitors effectively combated COVID‐19 with only few side effects (Table 2). PTC299, which is an orally bioavailable compound, is a potential anti‐COVID‐19 inhibitor of DHODH that inhibits the replication of SARS‐CoV‐2 and the induction of inflammatory cytokines [56, 57]. Our computational results indicated that DHODH is a promising antiviral target for COVID‐19, which is consistent with the results of previous reports [56, 57, 58, 59, 60]. We also discovered that 26 drugs in the DrugBank database [61] can inhibit DHODH. All of these drugs are regarded as potential candidates for drug repurposing to combat COVID‐19.

### VBR without viral lipids

We also reconstructed a cell‐specific GSMM of SARS‐CoV‐2‐infected lungs but with a VBR that did not include viral lipids. We then used this GSMM on the AVTD platform to identify antiviral genes and compared the results with those obtained from the aforementioned model, in which the VBR includes viral lipids. As presented in Table 2, 15 downregulated or knockout genes reduced the viral growth rate. Our computational results also revealed that five antiviral genes (Table 2) reduced the viral growth rate and achieved metabolic deviation grades that were nearly identical to those of the model in which the VBR included viral lipids. Therefore, we used STRING (https://string‐db.org/) and GeneCards (https://www.genecards.org/) to investigate the PPI networks encoded by the 15 aforementioned genes (Fig. 5B). The results indicated that eight genes were involved in nucleotide and pyrimidine metabolism, 11 genes were involved in glycolysis and glycosaminoglycan metabolism, and two genes were involved in cholesterol biosynthesis (the same antiviral targets obtained using the VBR with viral lipids). We also investigated whether the genes involved in glycerophospholipid or ceramide *de novo* biosynthesis served as antiviral targets. We used eight genes in both biosynthetic pathways (Table 2) on the AVTD platform to evaluate the performance of each target. These genes involved in glycerophospholipid and ceramide *de novo* biosynthesis are used to regulate the production of lipid components that serve as the building blocks of the viral membrane. However, we were unable to predict the VBGR because the VBR lacked lipid information. These results indicated that antiviral genes are underestimated if the VBR does not include the stoichiometry of lipids.

### Combination of antiviral targets

Drug combinations can be used to increase therapeutic efficacy and reduce toxicity [62]. The use of such combinations might increase the success rate of drug repurposing. However, the wet‐laboratory approach for identifying and validating effective combinations is limited by the excessive number of potential target combinations. In addition, conducting an enumerative search to identify combinations of two antiviral targets is time‐consuming because it requires computations of more than 4 million combinations to obtain optimal targets. Therefore, to identify two‐target combinations and reduce the computational burden, we developed two groups of candidates for the NHDE algorithm to generate searching individuals. The first candidate group comprised the 23 one‐target genes listed in Table 2, and the second group comprised other candidate genes from reduced encoding enzymes. This strategy substantially shortened the computational time and reduced the search space to approximately half a million possible combinations for the two candidate groups. We then used HV models that included and did not include the stoichiometry of lipids to identify two‐target combinations (18 combinations are listed in Table 3). We also identified many combinations for HV models that included (Table S7) and did not include the stoichiometry of lipids (Table S8) to diminish the VBGR.

**Table 3:** Optimal combinations of two‐target enzymes with a viral biomass reaction (VBR) that includes and not include the stoichiometry of lipids. The superscripts ♣ and ♥ indicate that an enzyme has duplicate enzymes and isozymes, respectively, in the model. * indicates a complex enzyme. VBGR and VATP represent viral biomass growth rate and ATP production rate of treated HV cells.

Genes	VBGR	VATP	$\eta_{\mathrm{CV}}^{\mathrm{TR}}$	$\eta_{\mathrm{MD}}^{\mathrm{TP}}$
**VBR includes the stoichiometry of lipids**				
(FH, UQCR11)	0	38	1	0.5771
(SULT2A1, PTPMT1)	0	38	1	0.5569
(CRLS1, FH)	0	38	1	0.556
(ACOX1, SLC2A13)	0	38	1	0.5559
(DHODH, MOXD1)	0	38	1	0.5525
(RPIA, (CTPS1, CTPS2)^♣^)	0	38	1	0.5524
(DHODH, SLC2A13)	0	38	1	0.5461
(UEVLD, CRLS1)	0	38	1	0.5451
(UMPS, DLST)	0	38	1	0.5432
(PTPMT1, (PGK1, PGK2, MIA3)^♣^)	0	38	1	0.5379
(CRLS1, (CTPS1, CTPS2)^♣^)	0	38	1	0.5376
(FUT1, PGS1)	0	38	1	0.5371
(PGS1, PTDSS1)	0	38	1	0.534
(SLC7A8, (CTPS1, CTPS2) ^♣^)	0	37.545	0.991	0.5684
(PGS1, (CTPS1, CTPS2) ^♣^)	0	36.802	0.9764	0.5688
(CRLS1, (GMPR2, GMPR)^♣^)	0	36.802	0.9764	0.4965
(HMGCS1, PTDSS1)	0	36.802	0.9764	0.481
(CRLS1, (BPGM, PGAM1, PGAM2)^♣^)	0	36.802	0.9764	0.4725
**VBR does not include the stoichiometry of lipids**				
((ELOVL2, ELOVL4, ELVOL5, ELOVL6)^♣^, (CTPS1, CTPS2)^♣^)	0	38	1	0.5644
(CAD, HAO1)	0	38	1	0.5356
(SLCO1B3, UMPS)	0	38	1	0.5291
(DHODH, ECHS1)	0	38	1	0.5166
(CAD, FH)	0	38	1	0.5155
(CRLS1, UMPS)	0	38	1	0.5142
(FH, UMPS)	0	38	1	0.5063
(CAD, (GMPR2, GMPR)^♣^)	0	36.802	0.9764	0.4891
(FUT1, (GAPDH, GPADHS)^♣^)	0.0248	38	0.9876	0.3651
(CRLS1, RPIA)	0.3952	38	0.8024	0.428
(CRLS1, PTDSS1)	1.5	38	0.25	0.2988
(CRLS1, PGS1)	1.5	38	0.25	0.2977
(KDSR, (CEPT1, SELENOI)^♥^)	1.5	38	0.25	0.2865
(PGS1, (SPTLC1, SPTLC2, SPTLC3)*)	1.5	38	0.25	0.2837
(KDSR, SLC2A13)	1.5	38	0.25	0.2809
((CEPT1, SELENOI)^♥^, PGS1)	1.5	38	0.25	0.2783
(PTPMT1, PTDSS1)	1.5	38	0.25	0.2766
(KDSR, (CEPT1, SELENOI)^♥^)	1.5	38	0.25	0.275

Our computational results revealed that two‐target combinations (Tables S7 and S8) achieved the complete elimination of viral biomass proliferation, the maximum ATP level (i.e., cell viability grade of approximately 100%), and a higher metabolic deviation grade than that of their single‐target counterparts (Table 2). We used eight single targets in glycerophospholipid and ceramide *de novo* biosynthesis (Table 2) as candidate genes to investigate their two‐target combinations. Our computational results indicated that the two‐target combinations of the HV model that did not include the stoichiometry of lipids were unable to reduce the VBGR because these two genes (from (CRLS1, PTDSS1) to (KDSR, (CEPT1, SELENOI)^♥^) in Table 3) were involved in glycerophospholipid and ceramide *de novo* biosynthesis, similar to the one‐target genes (Table 2). However, the HV model that included the stoichiometry of lipids completely inhibited viral biomass growth and improved the metabolic deviation grade, which resulted in only few side effects (Table 3). Therefore, antiviral genes are underestimated if the VBR does not include the stoichiometry of lipids.

## Conclusions

In this study, we used the genomic sequence and expression of the alpha variant of SARS‐CoV‐2 to reconstruct a cell‐specific GSMM for HV cells. We used a VBR in a reconstructed model that included and did not include the stoichiometry of lipids. Both reconstructed GSMMs for HV cells were applied on an AVTD platform to identify antiviral genes for treating COVID‐19, respectively. Our computational results revealed that the knockout or downregulation of genes involved in nucleotide and pyrimidine metabolism, glycolysis and glycosaminoglycan metabolism, and cholesterol biosynthesis reduces the biomass reaction rate of viral replication. We also discovered that the VBR that does not include the stoichiometry of lipids cannot be used to identify the genes involved in glycerophospholipid and ceramide *de novo* biosynthesis. These genes are used to regulate the production of lipid components that serve as the building blocks of the viral membrane.

## Conflict of interest

The authors declare no conflict of interest.

### Peer review

The peer review history for this article is available at https://www.webofscience.com/api/gateway/wos/peer‐review/10.1002/2211‐5463.13710.

## Author contributions

F‐SW was responsible for study conception and design and drafted the original manuscript. K‐LC reconstructed the model and performed the data analysis and database survey. All authors have read and approved the final manuscript.

## Supporting information


**File S1.** Computational procedures for solving AVTD platform and detailed description of the NHDE algorithm. Four figures and two tables are involved in Supplementary Table S1 to describe the computational procedures but not in the main manuscript. The figure and table legends are listed as follows.
**Fig. S1.** Work flowchart of the AVTD platform for identifying potential therapeutic antiviral targets to combat SARS‐CoV2.
**Fig. S2.** Computational procedures to obtain the optimal fluxes and metabolite flow rates provided as HV and HT templates.
**Fig. S3.** Evaluation of membership grades for fuzzy minimization, fuzzy maximization, fuzzy similarity, and fuzzy dissimilarity.
**Fig. S4.** Flowchart of the parallel search algorithm in NHDE.
**Table S1.** Basic operations for the original DE and NHDE algorithms.
**Table S2.** NHDE algorithm for iteratively selecting a set of candidate enzymes and to identify optimal targets.

**Table S3.** Cell‐specific genome‐scale metabolic models for host (HT) cells stored in Excel format.

**Table S4.** Cell‐specific genome‐scale metabolic models for host (HT) cells stored in XML format.

**Table S5.** Cell‐specific genome‐scale metabolic models for host–virus (HV) cells stored in Excel format.

**Table S6.** Cell‐specific genome‐scale metabolic models for host–virus (HV) cells stored in XML format.

**Table S7.** Optimal combinations of two‐target enzymes with a viral biomass reaction that includes the stoichiometry of lipids.
**Table S8.** Optimal combinations of two‐target enzymes with a viral biomass reaction that does not include the stoichiometry of lipids.

## Data Availability

The data that support the findings of this study are available in the [Link], [Link], [Link], [Link], [Link], [Link] of this article.
