# Supplementary material for: Cell‐specific genome‐scale metabolic modeling of SARS‐CoV‐2‐infected lung to identify antiviral enzymes
Source: FEBS Open Bio. 2023 Sep 30;13(12):2172–86. doi: 10.1002/2211-5463.13710 (PMC10699103; doi:10.1002/2211-5463.13710)
Supplement: Supplementary file 1 — File S1. Computational procedures for solving AVTD platform and detailed description of the NHDE algorithm. Four figures and two tables are involved in Supplementary Table S1 to describe the computational procedures but not in the main manuscript. The figure and table legends are listed as follows. Fig. S1. Work flowchart of the AVTD platform for identifying potential therapeutic antiviral targets to combat SARS‐CoV2. Fig. S2. Computational procedures to obtain the optimal fluxes and metabolite flow rates provided as HV and HT templates. Fig. S3. Evaluation of membership grades for fuzzy minimization, fuzzy maximization, fuzzy similarity, and fuzzy dissimilarity. Fig. S4. Flowchart of the parallel search algorithm in NHDE. Table S1. Basic operations for the original DE and NHDE algorithms. Table S2. NHDE algorithm for iteratively selecting a set of candidate enzymes and to identify optimal targets. [file FEB4-13-2172-s004.pdf]

Supplementary File S1:

Computational procedures for solving AVTD platform and detail description of the NHDE algorithm. Four figures and two tables are involved in the Supplementary File S1 to describe the computational procedures but not in the main manuscript. The figure and table legends are listed as follows:

Figure S1. Work flowchart of the AVTD platform for identifying potential therapeutic antiviral targets to combat SARS-CoV2

Figure S2. Computational procedures to obtain the optimal fluxes and metabolite flow rates provided as HV and HT templates.

Figure S3. Evaluation of membership grades for fuzzy minimization, fuzzy maximization, fuzzy similarity and fuzzy dissimilarity.

Figure S4. Flowchart of the parallel search algorithm in NHDE

Table S1. Basic operations for the original DE and NHDE algorithms

Table S2. The NHDE algorithm for iteratively selecting a set of candidate enzymes and to identify optimal targets.

The work flowchart of the AVTD platform is illustrated in Figure S1.

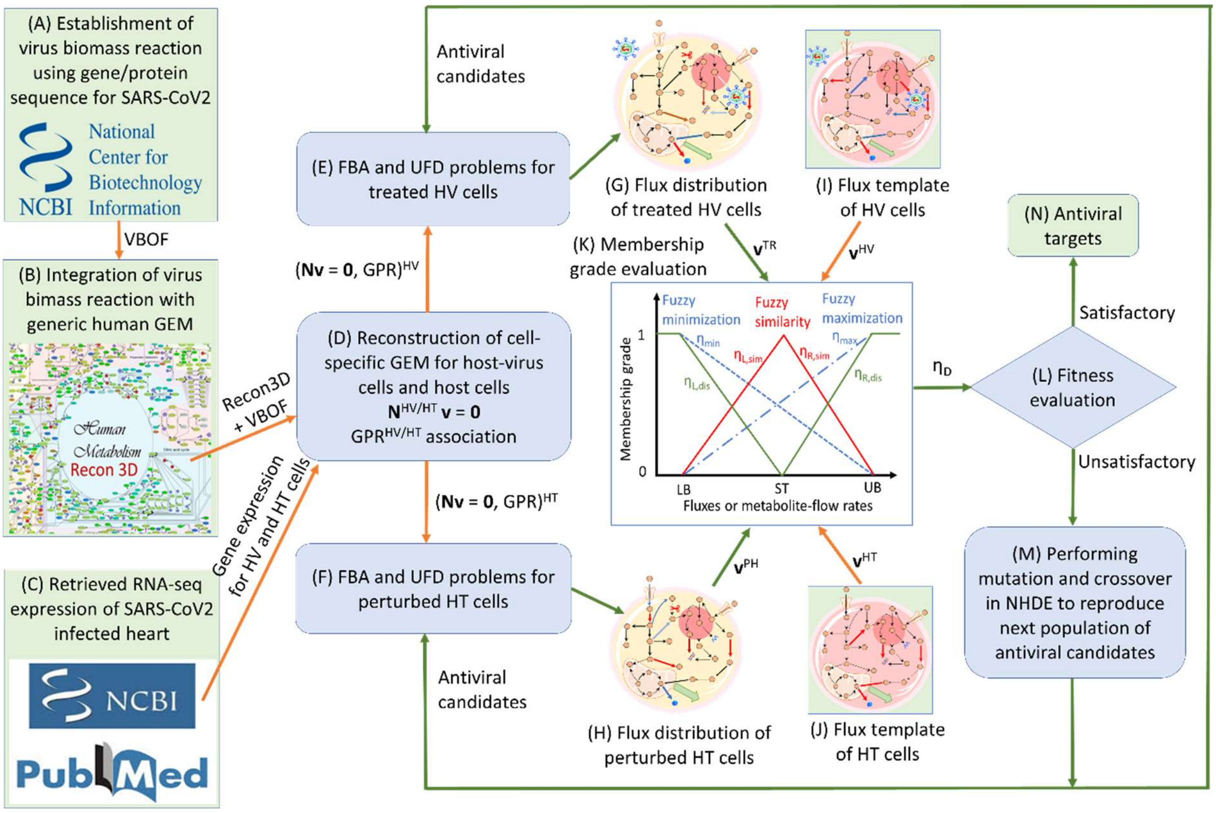

Figure S1. Work flowchart of the AVTD platform for identifying potential therapeutic antiviral targets to combat SARS-CoV2.

The preliminary steps for solving AVTD platform are to reconstruct each cell-specific genome-scale metabolic models for host-virus (HV) and healthy (HT) cells, and then to provide the templates for HV and HT models. The preliminary steps in Figure S1 are explained as the following three procedures.

Step A. We download the gene and protein sequence of the SARS-CoV-2 alpha variant could be downloaded from National Center for Biotechnology Information (NCBI) (https://www.ncbi.nlm.nih.gov/nuccore/) and to generate the stoichiometric coefficients of protein polymerization in the viral biomass reaction.

Step B. Integration of a generic human genome-scale metabolic network with the built viral biomass reaction to form a universal network.

Step C. We download RNA-seq expression for HV and HT cells to reconstruct cell-specific GSMM and the corresponding GPR association for HV and HT cells, respectively.

Step D. Reconstruction of cell-specific GSMM and its corresponding GPR association for HV and HT cells, respectively.

Step E and F. We build the constraint-based (CMB) model for HV and HT models in the inner optimization problems as follows.

$$
\begin{cases}
\text{Treated HV model:} \\
\text{FBA problem:} \qquad\qquad\qquad\qquad\qquad \text{UFD problem:} \\
\begin{cases}
\max\limits_{\mathbf{v}_{f/b}} v_{VBOF} \\
\text{subject to} \\
\quad \mathbf{N}^{HV}\left(\mathbf{v}_f - \mathbf{v}_b\right) = \mathbf{0} \\
\quad v_{f/b,i}^{LB,TR} \le v_{f/b,i} \le v_{f/b,i}^{UB,TR},\ i \in \Omega^{TR} \\
\quad v_{f/b,j}^{LB} \le v_{f/b,j} \le v_{f/b,j}^{UB},\ j \notin \Omega^{TR}
\end{cases}
\quad
\begin{cases}
\min\limits_{\mathbf{v}_{f/b}} \sum\limits_{k \in \Omega^{Int}} c_k^{HV}\left(v_{f,k} + v_{b,k}\right) \\
\text{subject to} \\
\quad \mathbf{N}^{HV}\left(\mathbf{v}_f - \mathbf{v}_b\right) = \mathbf{0} \\
\quad v_{f/b,i}^{LB,TR} \le v_{f/b,i} \le v_{f/b,i}^{UB,TR},\ i \in \Omega^{TR} \\
\quad v_{f/b,j}^{LB} \le v_{f/b,j} \le v_{f/b,j}^{UB},\ j \notin \Omega^{TR} \\
\quad v_{VBOF} \ge v_{VBOF}^{*}
\end{cases} \\
\text{Perturbed HT model:} \\
\text{FBA problem:} \qquad\qquad\qquad\qquad\qquad \text{UFD problem:} \\
\begin{cases}
\max\limits_{\mathbf{v}_{f/b}} v_{ATP} \\
\text{subject to} \\
\quad \mathbf{N}^{HT}\left(\mathbf{v}_f - \mathbf{v}_b\right) = \mathbf{0} \\
\quad v_{f/b,i}^{LB,TR} \le v_{f/b,i} \le v_{f/b,i}^{UB,TR},\ i \in \Omega^{TR} \\
\quad v_{f/b,j}^{LB} \le v_{f/b,j} \le v_{f/b,j}^{UB},\ j \notin \Omega^{TR}
\end{cases}
\quad
\begin{cases}
\min\limits_{\mathbf{v}_{f/b}} \sum\limits_{k \in \Omega^{Int}} c_k^{HT}\left(v_{f,k} + v_{b,k}\right) \\
\text{subject to} \\
\quad \mathbf{N}^{HT}\left(\mathbf{v}_f - \mathbf{v}_b\right) = \mathbf{0} \\
\quad v_{f/b,i}^{LB,TR} \le v_{f/b,i} \le v_{f/b,i}^{UB,TR},\ i \in \Omega^{TR} \\
\quad v_{f/b,j}^{LB} \le v_{f/b,j} \le v_{f/b,j}^{UB},\ j \notin \Omega^{TR} \\
\quad v_{ATP} \ge v_{ATP}^{*}
\end{cases}
\end{cases}
\tag{S1}
$$

where the stochiometric matrices, $\mathbf{N}^{HV}$ and $\mathbf{N}^{HT}$, for HV and HT models are reconstructed using Step (B)-(D) in Figure S1. Our previous study [Wang et al., 2022a, b] used the identical weighting factors, i.e.   for UFD problems. In the present study, the RNA-seq expressions for HV and HT cells are not only used to reconstruct cell-specific GSMMs but also to set the weighting factors   and   for UFD problems to obtain uniform flux distributions. The weighting factors depended on quartile confidence classification using the RNA-seq expression of each cell. The four groups of confidence reactions are assigned as follows:

$$
c_k^{HV/HT} = \begin{cases}
\dfrac{1}{4},\ k \in \text{high confidence} \\
\dfrac{1}{2},\ k \in \text{medium confidence} \\
\dfrac{3}{4},\ k \in \text{negativec confidence} \\
1,\ k \in \text{other confidence or non-gene-expression}
\end{cases}
\tag{S2}
$$

$v_{f/b,i}^{LB,TR}$ and $v_{f/b,i}^{UB,TR}$ denote the lower and upper bound of the regulated forward-backward fluxes depended on gene- or metabolite-centric approach for activation. The regulation bounds for the gene-centric approach can be expressed as follows:

Regulated bounds for z_i-th active gene/enzyme:

Up-regulation:

$$\begin{cases} (1-\delta)v_{f,i}^{basal} + \delta v_{f,i}^{UB} \le v_{f,i} \le v_{f,i}^{UB} \\ v_{b,i}^{LB} \le v_{b,i} \le (1-\delta)v_{b,i}^{basal} + \delta v_{b,i}^{LB};\ z_i \in \Omega^{TR} \end{cases}$$

Down-regulation:

$$\begin{cases} v_{f,i}^{LB} \le v_{f,i} \le (1-\delta)v_{f,i}^{basal} + \delta v_{f,i}^{LB} \\ (1-\delta)v_{b,i}^{basal} + \delta v_{b,i}^{UB} \le v_{b,i} \le v_{b,i}^{UB};\ z_i \in \Omega^{TR} \backslash \Omega^{IZ} \end{cases}$$

$$\begin{cases} (1-\varepsilon)v_{f,i}^{basal} \le v_{f,i} \le (1+\varepsilon)v_{f,i}^{basal} \\ (1-\varepsilon)v_{b,i}^{basal} \le v_{b,i} \le (1+\varepsilon)v_{b,i}^{basal};\ z_i \in \Omega^{TR} \cap \Omega^{IZ} \end{cases}$$

Knockout:

$$\begin{cases} v_{f,i} = 0 \\ v_{b,i} = 0;\ z_i \in \Omega^{TR} \backslash \Omega^{IZ} \end{cases}$$

$$\begin{cases} (1-\varepsilon)v_{f,i}^{basal} \le v_{f,i} \le (1+\varepsilon)v_{f,i}^{basal} \\ (1-\varepsilon)v_{b,i}^{basal} \le v_{b,i} \le (1+\varepsilon)v_{b,i}^{basal};\ z_i \in \Omega^{TR} \cap \Omega^{IZ} \end{cases} \quad \text{(S3)}$$

where $v_{f,i}^{basal}$ and $v_{b,i}^{basal}$ are the basal value of the ith forward-backward flux obtained from HV and HT templates; Ω^{IZ} is the set of reactions regulated by isozymes determined using the GPR associations, and δ is the modulation parameter determined by a nested hybrid differential evolution (NHDE) algorithm [Wang et al., 2022a]. A reaction catalyzed by isozymes remains around its basal level; thus, we set the flux ratio ε to 0.03 in this study to restrict the flux value. Metabolite-centric regulators modulate the synthesis reactions of the active metabolites. The LBs and UBs of modulated reactions for the ith active metabolite are restricted as follows:

Regulated bounds for the z_i-th active metabolite:

Up-regulation:

$$\begin{cases} (1-\delta)v_{f,j}^{basal} + \delta v_{f,j}^{UB} \le v_{f,j} \le v_{f,j}^{UB};\ j \in N_{ij} > 0 \text{ and } j \in \Omega^{rxn} \\ (1-\delta)v_{b,j}^{basal} + \delta v_{b,j}^{UB} \le v_{b,j} \le v_{b,j}^{UB};\ j \in N_{ij} < 0 \text{ and } j \in \Omega^{rev} \end{cases}$$

Down-regulation: (S4)

$$\begin{cases} v_{f,j}^{LB} \le v_{f,j} \le (1-\delta)v_{f,j}^{basal} + \delta v_{f,j}^{LB};\ j \in N_{ij} > 0 \text{ and } j \in \Omega^{rxn} \\ v_{b,j}^{LB} \le v_{b,j} \le (1-\delta)v_{b,j}^{basal} + \delta v_{b,j}^{LB};\ j \in N_{ij} < 0 \text{ and } j \in \Omega^{rev} \end{cases}$$

Knockout:

$$\begin{cases} v_{f,j} = 0;\ j \in N_{ij} > 0 \text{ and } j \in \Omega^{rxn} \\ v_{b,j} = 0;\ j \in N_{ij} < 0 \text{ and } j \in \Omega^{rev} \end{cases}$$

where N_{ij} is the stoichiometric coefficient of the ith metabolite and the jth reaction.

We have to provide HV and HT templates (Step I and J) for the AVTD platform for identifying antiviral targets. Clinical data of the fluxes and metabolite flow can be used as the HV and HT templates. However, genome-scale clinical data are currently not available. We use Eq.(S1) to

compute optimal fluxes and metabolite flow rates for HV and HT cells to provide as the templates. The computational procedures are expressed in Figure S2

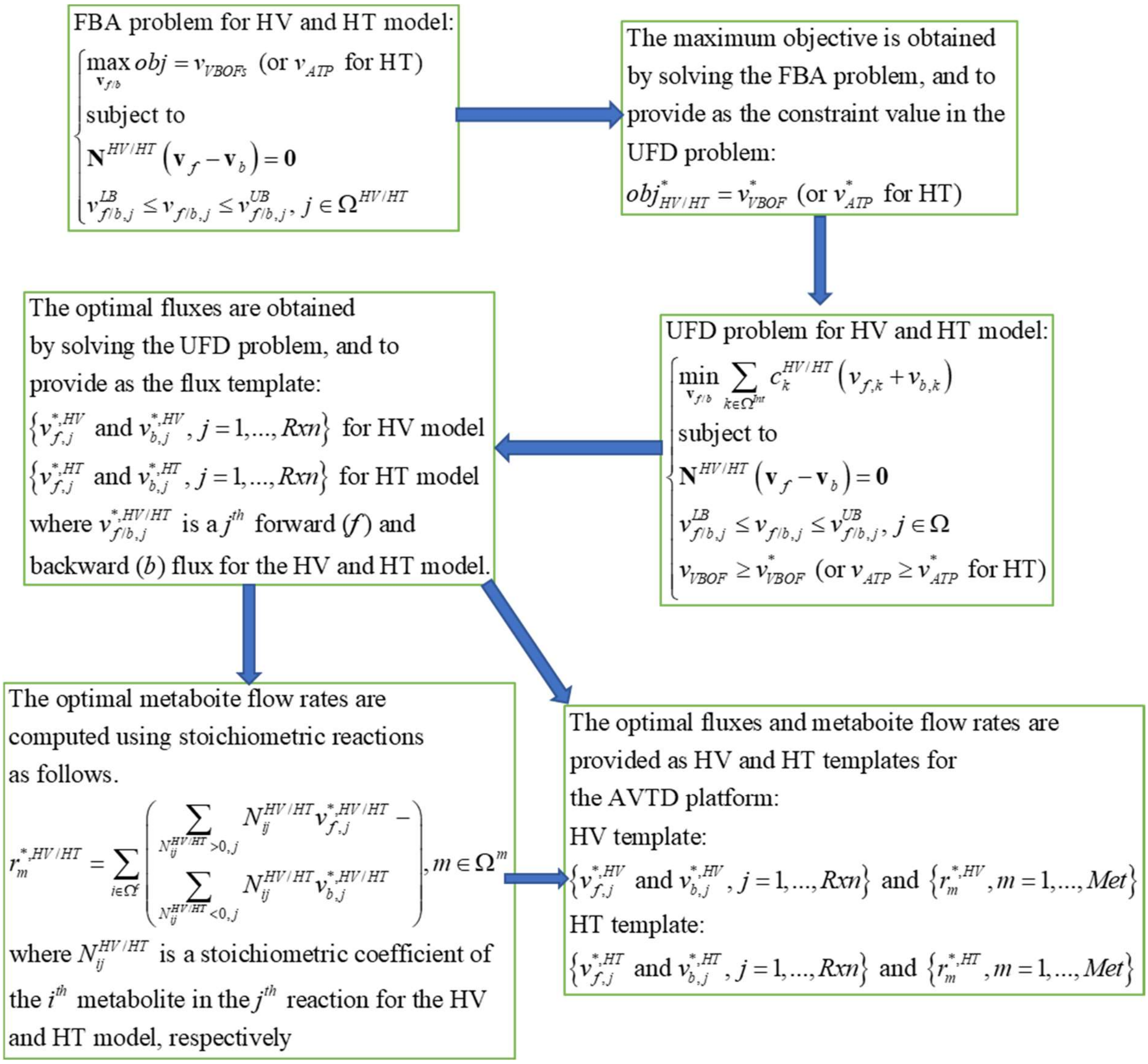

Figure S2. Computational procedures to obtain the optimal fluxes and metabolite flow rates provided as HV and HT templates.

Step G-N. The AVTD framework is a fuzzy multiobjective hierarchical optimization problem to mimic a wet-lab experiment to identify targets for treatment, and has discussed in our previous study [Wang et al., 2022b]. The design concept of the AVTD framework is described in Table 1.

The four goals in the outer optimization problem are explained as follows. The first goal is to evaluate fuzzy minimization ($\widetilde{\min}$) of the VBOF for the treated host-virus cells (denoted as TR) and cell-growth as follows:

$$\widetilde{\min_{\mathbf{z}}}\, v_{VBOF}^{TR} \approx 0 \tag{S5}$$

The second goal is to evaluate fuzzy maximization ($\widetilde{\max}$) of ATP production rate for TR and perturbed host cells (denoted as PH) cells as follows:

$$\begin{cases} \widetilde{\max_{\mathbf{z}}}\, v_{ATP}^{TR} \approx v_{ATP}^{\max} \\ \widetilde{\max_{\mathbf{z}}}\, v_{ATP}^{PH} \approx v_{ATP}^{\max} \end{cases} \tag{S6}$$

The third goal is to measure fuzzy similarity ($\widetilde{\text{similar}}$) of fluxes (v_j) and metabolite flow rates (r_m) of TR and PH cells relative to the healthy (HT) template as follows:

$$\begin{cases} \widetilde{\text{similar}}_{\mathbf{z}}\, v_j^{TR} \approx v_j^{HT} \\ \widetilde{\text{similar}}_{\mathbf{z}}\, r_m^{TR} \approx r_m^{HT} \\ \widetilde{\text{similar}}_{\mathbf{z}}\, v_j^{PH} \approx v_j^{HT} \\ \widetilde{\text{similar}}_{\mathbf{z}}\, r_m^{PH} \approx r_m^{HT} \end{cases} \tag{S7}$$

The fourth goal is to measure fuzzy dissimilarity ($\widetilde{\text{dissimilar}}$) that is used to evaluate the disparity of the fluxes and metabolite flow rates for TR and PH cells relative to those of the HV template, as expressed as follows:

$$\begin{cases} \widetilde{\text{dissimilar}}_{\mathbf{z}}\, v_j^{TR} \approx v_j^{HV} \\ \widetilde{\text{dissimilar}}_{\mathbf{z}}\, r_m^{TR} \approx r_m^{HV} \\ \widetilde{\text{dissimilar}}_{\mathbf{z}}\, v_j^{PH} \approx v_j^{HV} \\ \widetilde{\text{dissimilar}}_{\mathbf{z}}\, r_m^{PH} \approx r_m^{HV} \end{cases} \tag{S8}$$

In the aforementioned equations, the decision variables **z** represent the gene encoding enzymes as determined by a nest hybrid differential evolution (NHDE) algorithm (Described in the next section) for modulation. The fluxes $v_j^{HT/HV}$ and metabolite flow rates $r_m^{HT/HV}$ of the HT and HV templates can be obtained from clinical experimental data (if available). However, genome-scale clinical data are currently not available. Both templates computed from the HV and HT models as discussed in Figure S2 are provided for the computation.

The mth metabolite flow rate is computed using the following equations:

$$r_m = \sum_{i \in \Omega^c} \left(\sum_{N_{ij} > 0, j} N_{ij} v_{f,j} - \sum_{N_{ij} < 0, j} N_{ij} v_{b,j} \right), m \in \Omega^m \tag{S9}$$

where Ω^c is the set of species located in various compartments of HT and HV cells, and N_{ij} is a stoichiometric coefficient of the ith metabolite in the jth reaction of each GSMM. The forward flux $v_{f,j}$ and backward flux $v_{b,j}$ of the jth reaction are calculated by applying FBA and UFD models in the inner optimization problem as described in Step E.

The AVTD problem is transformed into a maximizing decision-making (MDM) problem through fuzzy set theory as illustrated in Figure 2 of the main text. The MDM problem is expressed as follows:

$$\begin{cases} \max\limits_{\mathbf{z}} \eta_D = \max\limits_{\mathbf{z}} \left(\eta_{CV}^{TR} + \min\left\{ \eta_{CV}^{TR}, \eta_{CV}^{PH}, \eta_{MD}^{TP} \right\} \right) \big/ 2 \\ \text{subject to inner optimization problems} \\ \text{1. FBA and UFD problems for treated HV cells} \\ \text{2. FBA and UFD problems for perturbed HT cells} \end{cases} \quad \text{(S10)}$$

where η_{CV}^{TR}, η_{CV}^{PH} and η_{MD}^{TP} denote as the cell viability grade of the TR model, cell viability grade of the PH model and metabolic deviation grade of the TR and PH models relative to their corresponding templates, and define as follows:

$$\eta_{CV}^{TR} = \left(\eta_{VBOF}^{TR} + \min\left\{ \eta_{VBOF}^{TR}, \eta_{ATP}^{TR} \right\} \right) \big/ 2 \quad \text{(S11)}$$

$$\eta_{CV}^{PH} = \eta_{ATP}^{PH} \quad \text{(S12)}$$

$$\eta_{MD}^{TP} = \frac{1}{2} \left(\frac{\left(\eta_{MD}^{TRHT} + \eta_{MD}^{PHHT} + \eta_{MD}^{TRHV} + \eta_{MD}^{PHHV} \right)}{4} + \min\left\{ \eta_{MD}^{TRHT}, \eta_{MD}^{PHHT}, \eta_{MD}^{TRHV}, \eta_{MD}^{PHHV} \right\} \right) \quad \text{(S12)}$$

The membership functions, η_{VBOF}^{TR} and η_{ATP}^{TR}, in Eq.(S11) use to represent fuzzy minimization and maximization for the TR model in Eqs.(S5) and (S6). Similarly, the membership function, η_{ATP}^{PH}, attribute to fuzzy optimization for the PH model in Eqs.(S5) and (S6). The membership grades for fuzzy minimization and fuzzy maximization are expressed in Figure S3. Both fluxes and metabolite flow rates for TR and PH models are used to compute the corresponding metabolic deviation grades through two-sided membership functions (Figure S3). The grades are then used to compute overall metabolic deviation grades of fuzzy similarity (η_{MD}^{TRHT} and η_{MD}^{PHHT}) relative to the HT template and fuzzy dissimilarity (η_{MD}^{TRHV} and η_{MD}^{PHHV}) relative to the HV template, respectively.

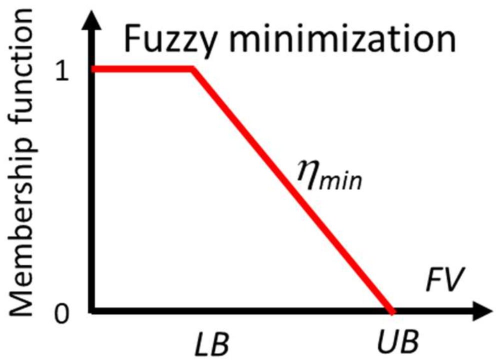

$$\eta_{\min} = \begin{cases} 1, & \text{if } FV < LB \\ \dfrac{UB - FV}{UB - LB}, & \text{if } LB \le FV \le UB \\ 0, & \text{if } FV > UB \end{cases}$$

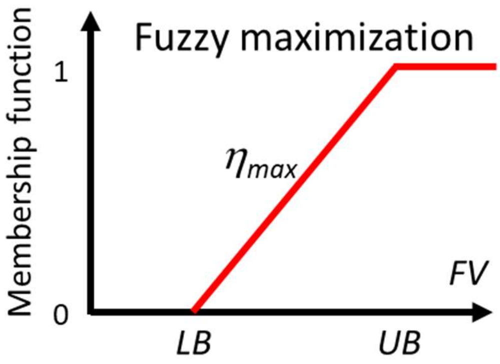

$$\eta_{\max} = \begin{cases} 0, & \text{if } FV < LB \\ \dfrac{FV - LB}{UB - LB}, & \text{if } LB \le FV \le UB \\ 1, & \text{if } FV > UB \end{cases}$$

The flux *FV* is obtained by solving Eq.(S1), and the lower bound *LB* and the upper bound *UB* are provided from HV and HT templates from Figure S2.

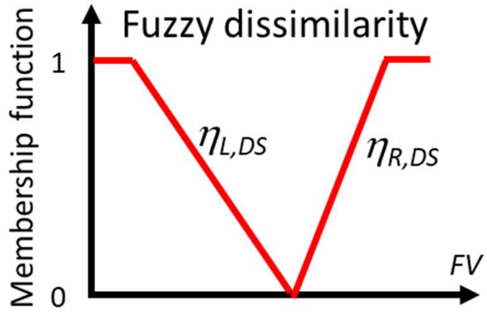

$$\eta_{L,DS}(FV) = \begin{cases} 1, & \text{if } FV < LB \\ \dfrac{ST - FV}{ST - LB}, & \text{if } LB \le FV \le ST \\ 0, & \text{if } FV > ST \end{cases}$$

$$\eta_{R,DS}(FV) = \begin{cases} 0, & \text{if } FV < ST \\ \dfrac{FV - ST}{UB - ST}, & \text{if } ST \le FV \le UB \\ 1, & \text{if } FV > UB \end{cases}$$

$$\eta_{DS} = \min\left\{\max\left\{\eta_{L,DS}, \eta_{R,DS}, 0\right\}, 1\right\}$$

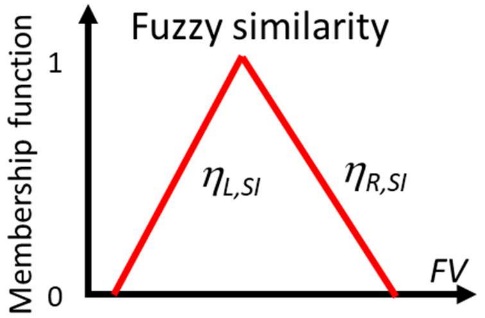

$$\eta_{L,SI} = \begin{cases} 0, & \text{if } FV < ST \\ \dfrac{FV - LB}{ST - LB}, & \text{if } LB \le FV \le ST \\ 1, & \text{if } FV > ST \end{cases}$$

$$\eta_{R,SI} = \begin{cases} 1, & \text{if } FV < ST \\ \dfrac{UB - FV}{UB - ST}, & \text{if } ST \le FV \le UB \\ 0, & \text{if } FV > UB \end{cases}$$

$$\eta_{SI} = \max\left\{\min\left\{\eta_{L,SI}, \eta_{R,SI}, 1\right\}, 0\right\}$$

FV denotes as a flux of ith reaction or a metabolite flow rate of ith metabolite that is obtained by solving Eq.(S1), and ST denotes as the optimal value of HV or HT templates obtained from Figure S2. The lower bound LB and the upper bound UB are provided from HV and HT templates as follows: LB = ST/4 and UB = 4ST.

Figure S3. Evaluation of membership grades for fuzzy minimization, fuzzy maximization, fuzzy similarity and fuzzy dissimilarity.

Introduction to Nested Hybrid Differential Evolution (NHDE)

The antiviral target discovery (AVTD) platform can be formulated as a fuzzy multi-objective hierarchical optimization problem. The AVTD platform can be transformed into a maximizing decision-making (MDM) problem by using fuzzy set theory to derive Pareto solutions as shown in Figure S1. The existence and limitation of the transformation have proved in Wang, et al. (2022a). The MDM problem is rewritten as the following simplified formulation for easily explaining the NHDE algorithm.

$$
\begin{cases}
\text{Outer optimization problem:} \\
\quad \max f(\mathbf{x},\mathbf{z}) \\
\text{subject to the inner optimization problems:} \\
\begin{cases}
\text{FBA problem} \\
\max\limits_{\mathbf{x}} obj = \mathbf{c}^T\mathbf{x} \\
\text{subject to} \\
\quad \mathbf{Ax} = \mathbf{0} \\
\quad \mathbf{x}_{LB} \le \mathbf{x} \le \mathbf{x}_{UB}, \mathbf{z} \notin \Omega^{TR} \\
\quad \mathbf{x}_{LB}^{TR} \le \mathbf{x} \le \mathbf{x}_{UB}^{TR}, \mathbf{z} \in \Omega^{TR} \\
\quad \mathbf{x} \ge \mathbf{0}
\end{cases}
\begin{cases}
\text{UFD problem} \\
\min\limits_{\mathbf{x}} \sum c_k x_k \\
\text{subject to} \\
\quad \mathbf{Ax} = \mathbf{0} \\
\quad \mathbf{x}_{LB} \le \mathbf{x} \le \mathbf{x}_{UB}, \mathbf{z} \notin \Omega^{TR} \\
\quad \mathbf{x}_{LB}^{TR} \le \mathbf{x} \le \mathbf{x}_{UB}^{TR}, \mathbf{z} \in \Omega^{TR} \\
\quad \mathbf{c}^T\mathbf{x} \ge \mathbf{c}^T\mathbf{x}^* \\
\quad \mathbf{x} \ge \mathbf{0}
\end{cases}
\end{cases}
$$

The inner optimization problem consists of two linear programming problems, which is a sequential relationship.

The NHDE algorithm is a stochastic optimization based on hybrid differential evolution (Chiou, 1997), which was extended from the original DE algorithm (Storn and Price, 1996; Storn and Price, 1997). The basic operations of original DE and modified NHDE are shown in Table S1. The detailed procedures have discussed by Wang (2017).

Table S1. Basic operations for the original DE and NHDE algorithms

Original DE	Modified NHDE
1. Representation and initialization	1. Representation and initialization
2. Mutation	2. Mutation with rounding operation
3. Crossover operation	3. Crossover operation
4. Selection and evaluation	4. Restriction operation
5. Repeat steps 2 to 4	5. Selection and evaluation
	6. Solve LP/QP problems for each candidate gene
	7. Compute fitness for each feasible design
	8. Migration operation performed naturally or enforced if necessary
	9. Repeat steps 2 to 6

The computational procedures of NHDE are listed in Table S2. NHDE is a parallel direct search algorithm (as shown in Figure S4) that utilizes a population of N_p individuals (enzymes) to find an optimal solution. The initialization process randomly generates N_p individuals to cover the entire search space uniformly. Each individual in the population consists of a set of enzymes that are selected to be modulated.

The mutation operator of NHDE adopted from DE was an essential component compared with other evolutionary algorithms. Different from conventional evolutionary algorithms, the mutation operation of DE/NHDE uses the difference between two or four randomly chosen individuals as an evolutionary direction. The ith mutant individual $(\mathbf{z}^G)_i$ in generation G is obtained through the difference of two or four random individuals as expressed in the following form:

$$(\mathbf{z}^G)_i = \text{INT}\left\{(\mathbf{z}^G)_p + \rho^G\left[(\mathbf{z}^G)_j - (\mathbf{z}^G)_k + (\mathbf{z}^G)_l - (\mathbf{z}^G)_m\right]\right\}, i = 1,\ldots,N_p$$

where random indices $j,k,l,m \in \{1,\ldots,N_p\}$ are mutually different. The operator INT in the equation is used to rounding the real vector into the integer vector. In DE, the differential mutation factor $\rho^G \in [0, 1.2]$ is fixed and set by the user to obtain faster convergence. This factor is used to control the step length along the searching direction. A random mutation factor was used in NHDE to obtain more diversified individuals. NHDE also includes an additional mutation strategy that applying a linear crossover for the ith individual and the best individual $(\mathbf{z}^G)_b$ to generate the parent individual. The parent individual is therefore expressed as follows:

$$(\mathbf{z}^G)_p = \rho_p^G(\mathbf{z}^G)_b + (1-\rho_p^G)(\mathbf{z}^{G-1})_i$$

where the factor ρ_p^G is a random number between zero and one generated by a uniform distribution generator, and $(\mathbf{z}^{G-1})_i$ indicates the ith mutant individual in the previous generation. The mutation operation may cause the mutant individual escape from the search domain. The mutation operation may cause the mutant individual to escape the search domain (i.e., bounds are violated). If this occurs, it is replaced by a random number within the lower and upper bounds of the particular decision variable, thus restricting to the search domain. The choice of mutation factor for DE/NHDE is heuristic and random. When population diversity is low, candidate individuals rapidly cluster together such that the individuals cannot be further improved, and premature convergence occurs. Similar to conventional evolutionary algorithms, the local population diversity could be increased by using a crossover operation such as a binomial crossover.

NHDE use the difference between two or four mutually independent individuals to determine the direction of search and obtain a mutant individual. This differential mutation converges quickly so that most individuals cluster around the best candidate individual in some generations. Consequently, the population diversity and exploration capability diminish and clustered individuals are unable to reproduce more diversified individuals through the mutation operation because the weighted difference is nearly zero. The recombination of mutant individuals and their clustered parents further prevents the reproduction of a diversified population. Therefore, all individuals quickly cluster together and superior individuals cannot be generated through mutation and crossover operations.

The migration operation of the NHDE algorithm is used to help individuals escape from the local cluster, but this operation is performed only if the population diversity falls below a desired level. The degree of population diversity ζ is introduced to check whether the migration operation should be performed. Each element of the ith individual $(\mathbf{z}^G)_i$ in generation G is referred to as a gene of the individual, and the gene diversity index dz_{ji} is given by

$$dz_{ji} = \begin{cases} 0, & \text{if } z_{ji}^G = z_{jb}^G,\ j = 1,\dots,n; i = 1,\dots,N_p; i \neq b \\ 1, & \text{otherwise,} \end{cases}$$

where z_{ji}^G and z_{jb}^G are the jth gene of the ith and best individual at the Gth generation, respectively. dz_{ji} is set to zero if the jth gene of the ith individual is identical to the best gene; otherwise it is set to one (Chiou and Wang, 1999; Liao, et al., 2001). ζ is defined as the ratio of total gene diversities to the total number of genes other than those of the best individual:

$$\zeta = \frac{\sum_{i=1, i\neq b}^{N_p} \sum_{j=1}^{n} dz_{ji}}{n(N_p - 1)}$$

The value of population diversity degree ranges between zero and one. A value of zero implies that all of the genes are clustered around the best individual. On the other hand, a value of one indicates that current candidate individuals are a completely diversified population. The desired tolerance for population diversity is assigned by the user. A tolerance value of zero implies that the migration operation in NHDE is switched off, and one implies that the migration operation is performed at every generation. Consequently, the user can set a tolerance value for population diversity degree, $\varepsilon \in (0, 1)$. If ζ is smaller than ε, then NHDE performs migration operations to regenerate a new population in order to escape from a local point; otherwise, NHDE suspends the migration operation and maintains a constant search direction toward finding a new solution.

Table S2. The NHDE algorithm for iteratively selecting a set of candidate enzymes and to identify optimal targets.

NHDE
1. Representation and initialization<br>$(\mathbf{z}^0)_i = \text{uniformInt}(\mathbf{z}^{\min}, \mathbf{z}^{\max}), i = 1,..., N_p$<br>Each individual is generated by an integer random number between $\mathbf{z}_{\min}$ and $\mathbf{z}_{\max}$ with uniform distribution
2. Mutation with rounding operation<br>$(\hat{\mathbf{z}}^G)_i = \text{INT}\left\{(\mathbf{z}^G)_p + \rho^G\left[(\mathbf{z}^G)_j - (\mathbf{z}^G)_k + (\mathbf{z}^G)_l - (\mathbf{z}^G)_m\right]\right\}$
3. Crossover operation<br>$z_{ji}^G = \begin{cases} z_{ji}^{G-1}, & \text{if a random number} > C_R \\ \hat{z}_{ji}^G, & \text{otherwise}, j = 1,...,n; i = 1,..., N_p \end{cases}$
4. Restriction operation<br>$z_{ji}^G = \begin{cases} z_{ji}^G, & z_{ji}^G \in [z_j^{\min}, z_j^{\max}] \\ \text{uniformInt}(z_j^{\min}, z_j^{\max}), & z_{ji}^G \notin [z_j^{\min}, z_j^{\max}] \end{cases}$
5. Selection and evaluation<br>(a) For each enzyme, solve the inner LP/QP problem by LP/QP solver, respectively<br>(b) Compute fitness for each feasible solution<br>$\mathit{fitness} = f(\mathbf{x}, \mathbf{z}) + \text{penalty}$
6. Migration operation performed naturally or enforced if necessary<br>$(\mathbf{z}^G)_i = \text{uniformInt}(\mathbf{z}^{\min}, \mathbf{z}^{\max}), \text{if } \zeta \le \varepsilon = [0,1]$
7. Repeat steps 2 to 6

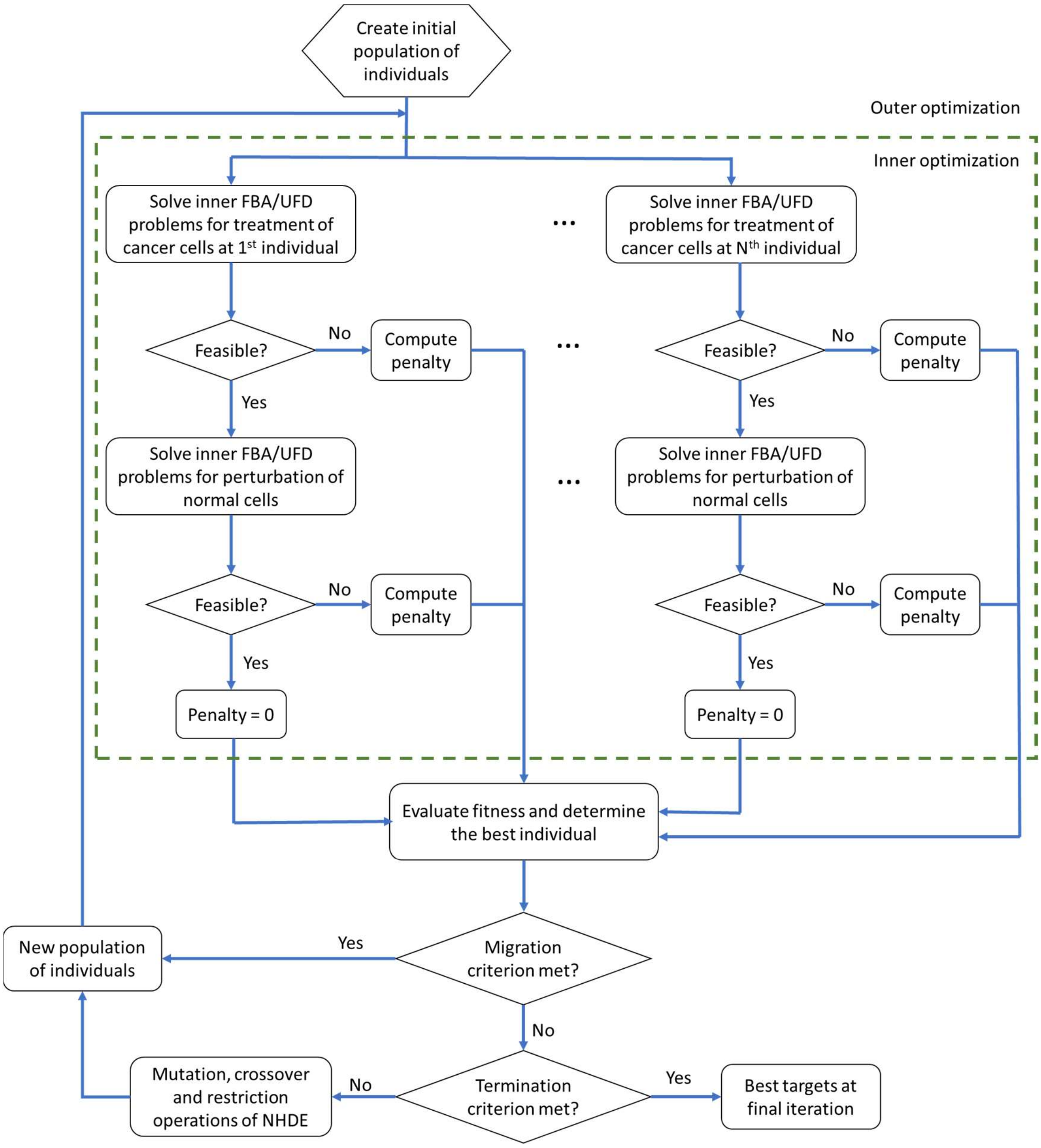

Figure S4. Flowchart of the parallel search algorithm in NHDE

References

1. Wang, F.S., Wang, T.Y. and Wu, W.H. (2022a) Fuzzy multiobjective hierarchical optimization with application to identify antienzymes of colon cancer cells, Journal of the Taiwan Institute of Chemical Engineers, 132, 10412. (doi.org/10.1016/j.jtice.2021.10.021)
2. Wang, F.S., Chen, K.L. and Chu, S.W. (2022b) Human/SARS-CoV-2 genome-scale metabolic modeling to discover potential antiviral targets for COVID-19, Journal of the Taiwan Institute of Chemical Engineers, 133, 104273. (doi.org/10.1016/j.jtice.2022.104273)
3. Chiou, J.P. and Wang, F.S. (1999) Hybrid method of evolutionary algorithms for static and dynamic optimization problems with application to a fed-batch fermentation process, Computers & Chemical Engineering, 23, 1277-1291. (doi.org/10.1016/S0098-1354(99)00290-2)
4. Storn, R. and Price, K. (1996) Minimizing the real functions of the ICEC'96 contest by differential evolution. Evolutionary Computation, 1996., Proceedings of IEEE International Conference on. IEEE, Nagoya, pp. 842 - 844. (doi:10.1109/ICEC.1996.542711)
5. Storn, R. and Price, K. (1997) Differential evolution - A simple and efficient heuristic for global optimization over continuous spaces, Journal of Global Optimization, 11, 341-359. (doi.org/10.1023/A:1008202821328)
6. Wang, F.S. (2017) Nested differential evolution for mixed-integer bi-level optimization for genome-scale metabolic networks, Ch.12, in Differential evolution in chemical engineering edited by Rangaiah and Sharma, World Scientific. (doi.org/10.1142/10379)
